# Supplementary material for: Halosmysin A, a Novel 14-Membered Macrodiolide Isolated from the Marine-Algae-Derived Fungus Halosphaeriaceae sp
Source: Mar Drugs. 2020 Jun 18;18(6):320. doi: 10.3390/md18060320 (PMC7344848; doi:10.3390/md18060320)

# Supplementary Materials

# Halosmysin A, a novel 14-membered macrodiolide isolated from the marine-alga-derived fungus *Halosphaeriaceae sp.*

**Takeshi Yamada[1,*], Haruka Kogure[1], Minami Kataoka[1], Takashi Kikuchi[1], and Tomoya Hirano**

## The Table of Contents


| | | |
|---|---|---|
| **Table S1** | **Spectral data including 2D NMR data for 1** | **1** |
| **Figure S1** | **14-membered macrodiolides associated to halosmysin A** | **2** |
| **Figure S2** | **$^{1}$H NMR spectrum of 1 in $CDCl_3$** | **3** |
| **Figure S3** | **$^{13}$C NMR spectrum of 1 in $CDCl_3$** | **9** |
| **Figure S4** | **$^{1}$H-$^{1}$H COSY of 1** | **11** |
| **Figure S5** | **NOESY of 1** | **14** |
| **Figure S6** | **HMQC of 1** | **17** |
| **Figure S7** | **HMBC of 1** | **21** |
| **Figure S8** | **FABMS of 1** | **26** |
| **Figure S9** | **IR spectrum of 1** | **29** |

Table S1 NMR spectral data of **1** in $CDCl_3$ [d]

| Position | $\delta_H$ [a] | | J/Hz | $^1H$-$^1H$ COSY | NOESY [b] | $\delta_C$ | | HMBC (C) [c] |
|---|---|---|---|---|---|---|---|---|
| 2 | | | | | | 165.6 | (s) | |
| 3 | 5.64 | d | 16.2 (4) | 4 | 5β, 9 | 125.3 | (d) | 2, 5 |
| 4 | 6.78 | ddd | 16.2 (3), 10.2 (5β), 6.0 (5α) | 3, 5α, 5β | 6 | 143.5 | (d) | 2, 5 |
| 5α | 2.48 | ddd | 13.2 (5β), 6.0 (4), 1.2 (6) | 4, 5β | 15 | 39.0 | (t) | 3, 4 |
| 5β | 2.23 | ddd | 13.2 (5α), 13.2 (6), 10.2 (4) | 4, 5α, 6 | 3, 15 | | | 3, 4, 6, 15 |
| 6 | 5.30 | dqd | 13.2 (5β), 6.0 (15), 1.2 (5α) | 5β, 6-$CH_3$ | 4 | 69.9 | (d) | |
| 8 | | | | | | 168.9 | (s) | |
| 9 | 3.38 | d | 3.0 (10) | 10 | 3, 2', 7'A, 7'B | 51.9 | (d) | 8, 10, 11, 6' |
| 10 | 4.57 | d | 3.0 (9) | 9 | 13β, 2' | 51.0 | (d) | 8, 11, 3' |
| 11 | | | | | | 208.2 | (s) | |
| 12 | 4.55 | d | 7.8 (13α) | 13α | S-$CH_3$ | 75.6 | (d) | 11, 13, 14 |
| 13α | 1.96 | ddd | 14.4 (13β), 7.8 (12), 3.0 (14) | 12, 13β | 16 | 37.4 | (t) | 11, 12 |
| 13β | 2.64 | ddd | 14.4 (13α), 12.0 (14), 1.2 (12) | 13α, 14 | 10, 16 | | | 14, 16 |
| 14 | 5.23 | dqd | 12.0 (13β), 6.0 (16), 3.0 (13α) | 13β, 14-$CH_3$ | | 65.5 | (d) | |
| 15 | 1.44 | d | 6.0 (6) | 6 | 5α, 5β, 7'A | 20.9 | (q) | 5, 6 |
| 16 | 1.26 | d | 6.0 (14) | 14 | 13α, 13β | 20.2 | (q) | 13, 14 |
| 12-OH | Not observed | | | | | | | |
| 1' (NH) | 5.73 | s | | | 7'A, 9' | | | 3', 6' |
| 2' | | | | | | 165.7 | (s) | |
| 3' | | | | | | 68.6 | (s) | |
| 4' (NH) | 5.95 | s | | | 9, 10, S-$CH_3$, | | | 2', 6' |
| 5' | | | | | | 170.9 | (s) | |
| 6' | | | | | | 63.7 | (s) | |
| 7'A | 2.83 | d | 14.4 (7'B) | 7'B | 9, 15, 1', 9' | 33.0 | (t) | 9, 5', 6', 8', 9' |
| 7'B | 3.88 | d | 14.4 (7'A) | 7'A | 9, 9' | | | 5', 6', 8', 9' |
| 8' | | | | | | 125.5 | (s) | |
| 9' | 6.83 | d | 9.0 (10') | 10' | 5', 7'A, 7'B | 131.7 | (d) | 7', 8', 11' |
| 10' | 7.10 | d | 9.0 (9') | 9' | 12' | 115.2 | (d) | 8', 11' |
| 11' | | | | | | 158.5 | (s) | |
| 12' | 4.47 | d | 7.2 (13') | 13' | 10', 16' | 64.8 | (t) | 11', 13', 14' |
| 13' | 5.47 | br t | 7.2 (12') | 12' | 15' | 119.5 | (d) | 15', 16' |
| 14' | | | | | | 138.4 | (s) | |
| 15' | 1.79 | s | | | 13' | 25.8 | (q) | 13', 14', 16' |
| 16' | 1.74 | s | | | 12' | 18.2 | (q) | 13', 14', 15' |
| S-$CH_3$ | 2.25 | s | | | 12, 2' | 13.0 | (q) | 3' |

a $^1H$ chemical shift values (δ ppm from $SiMe_4$) followed by multiplicity and then the coupling constants (*J*/Hz). Figures in parentheses indicate the proton coupling with that position. b The correlations with geminal and vicinal protones are removed. c Long range $^1H$-$^{13}C$ correlations from H to C observed in the HMBC experiment. d 600 MHz ($^1H$ NMR), 150 MHz ($^{13}C$ NMR)

## Figure S1 14-membered macrodiolides associated to halosmysin A

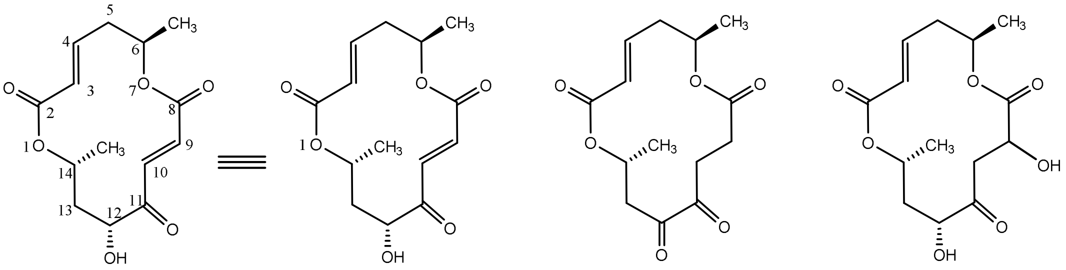

colletoketol | grahamimycin A | grahamimycin $A_1$ | grahamimycin B

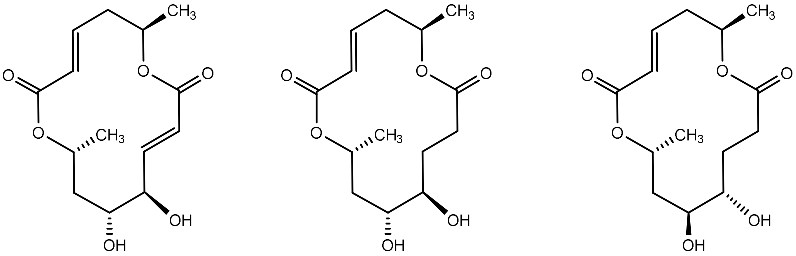

(6*R*,11*R*,12*R*,14*R*)-colletodiol | 9,10-dihydro-(6*R*,11*R*,12*R*,14*R*)-colletodiol | 9,10-dihydro-(6*R*,11*S*,12*S*,14*R*)-colletodiol

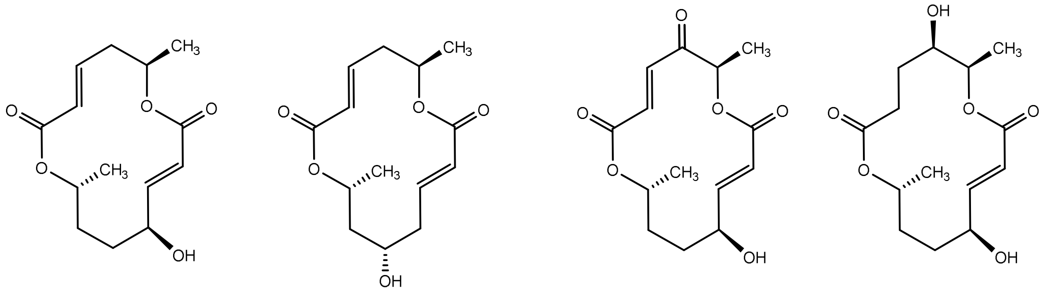

colletallol | colletol | acremonol | acremodiol

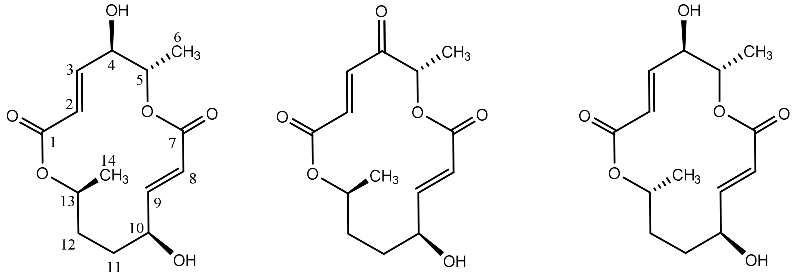

clonostachydiol | 4-keto-clonostachydiol | cordybislactone

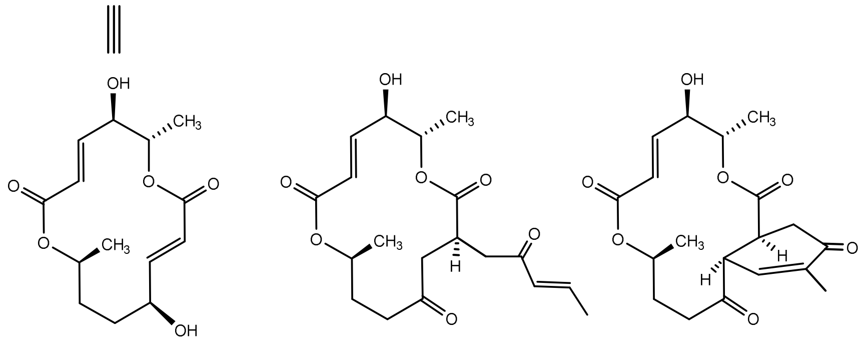

acaudiol A | acaulone A | acaulone B

## Figure S2 $^{1}H$ NMR spectrum of 1 in $CDCl_3$

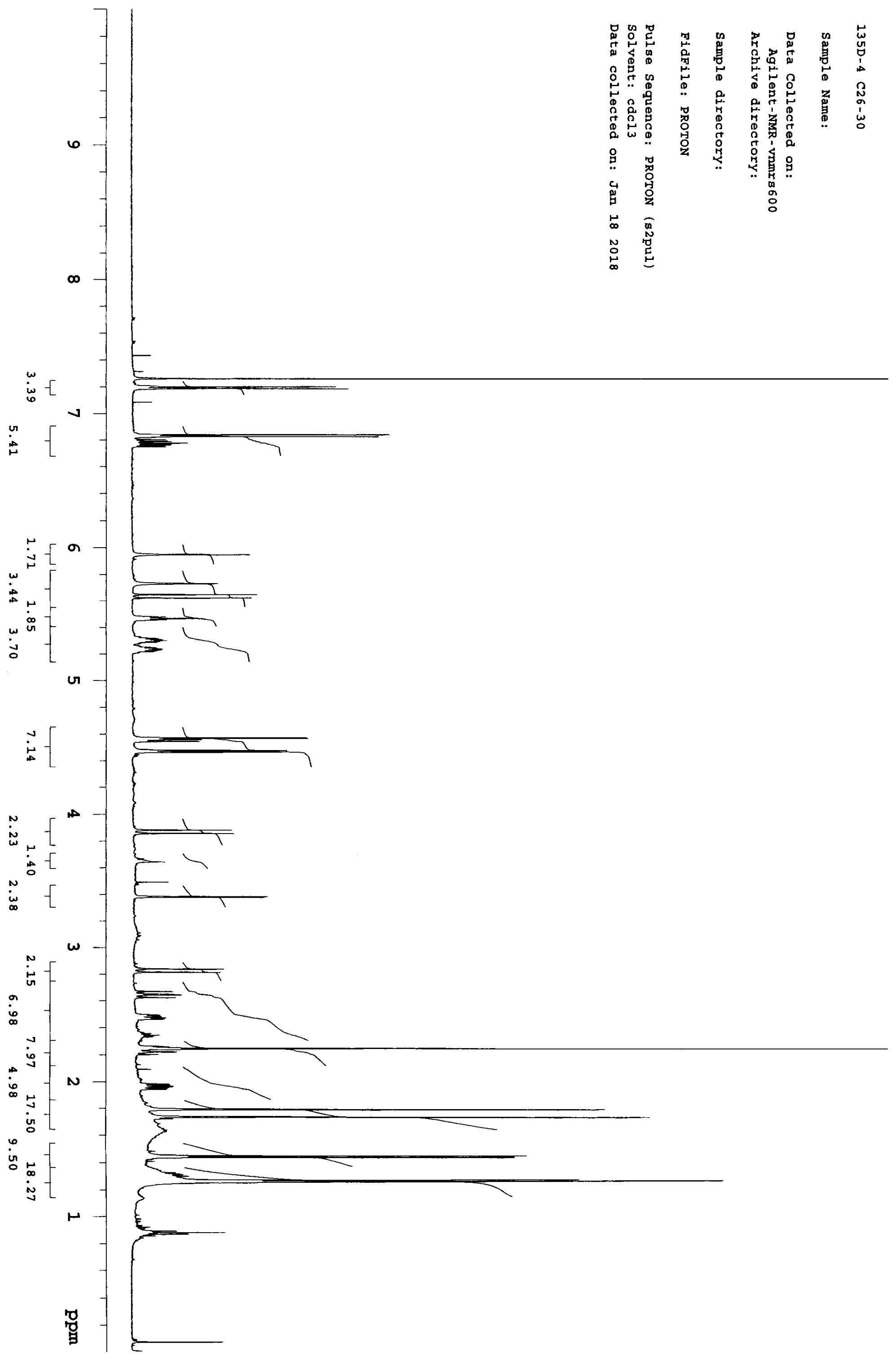

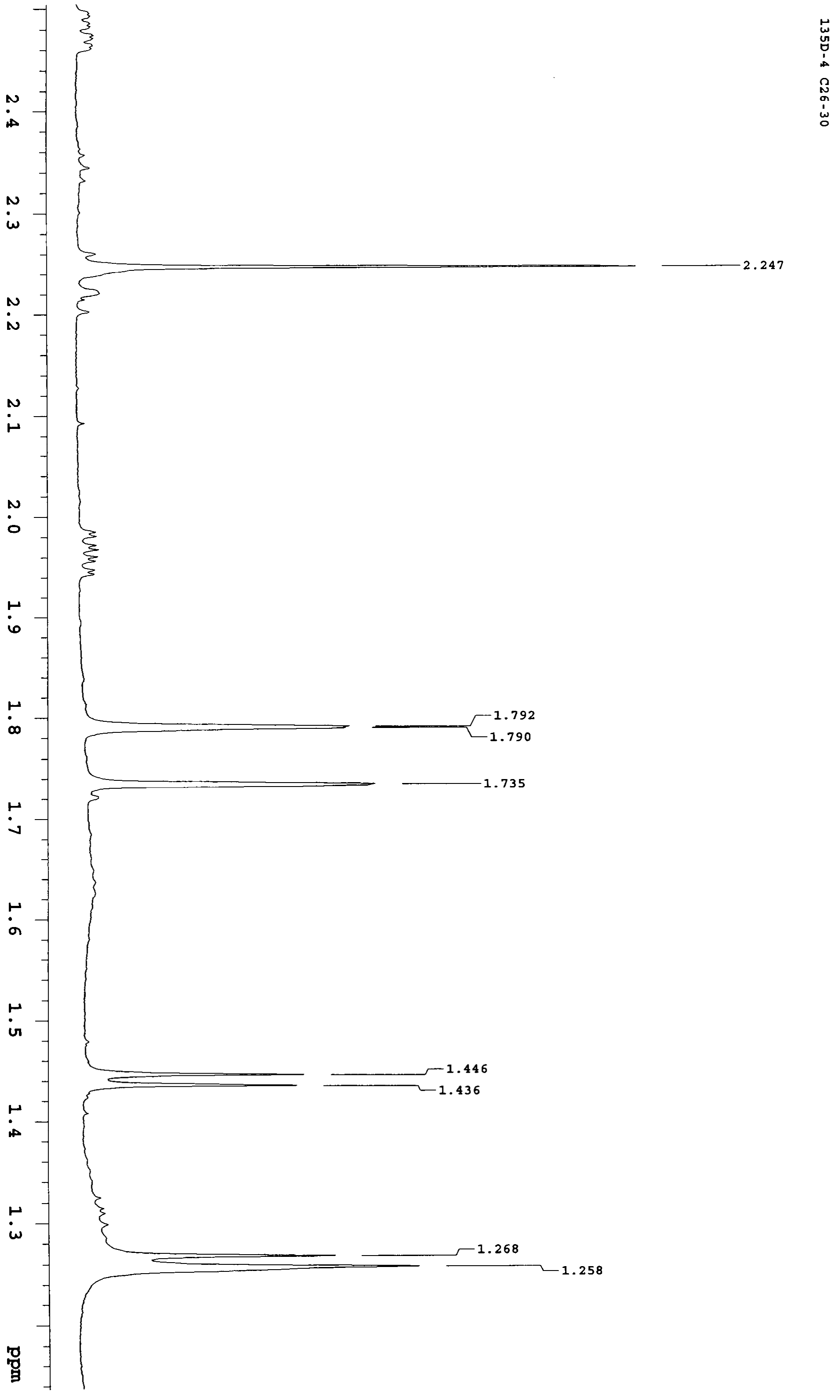

135D-4 C26-30
2.247
1.792
1.790
1.735
1.446
1.436
1.268
1.258
2.4
2.3
2.2
2.1
2.0
1.9
1.8
1.7
1.6
1.5
1.4
1.3
ppm

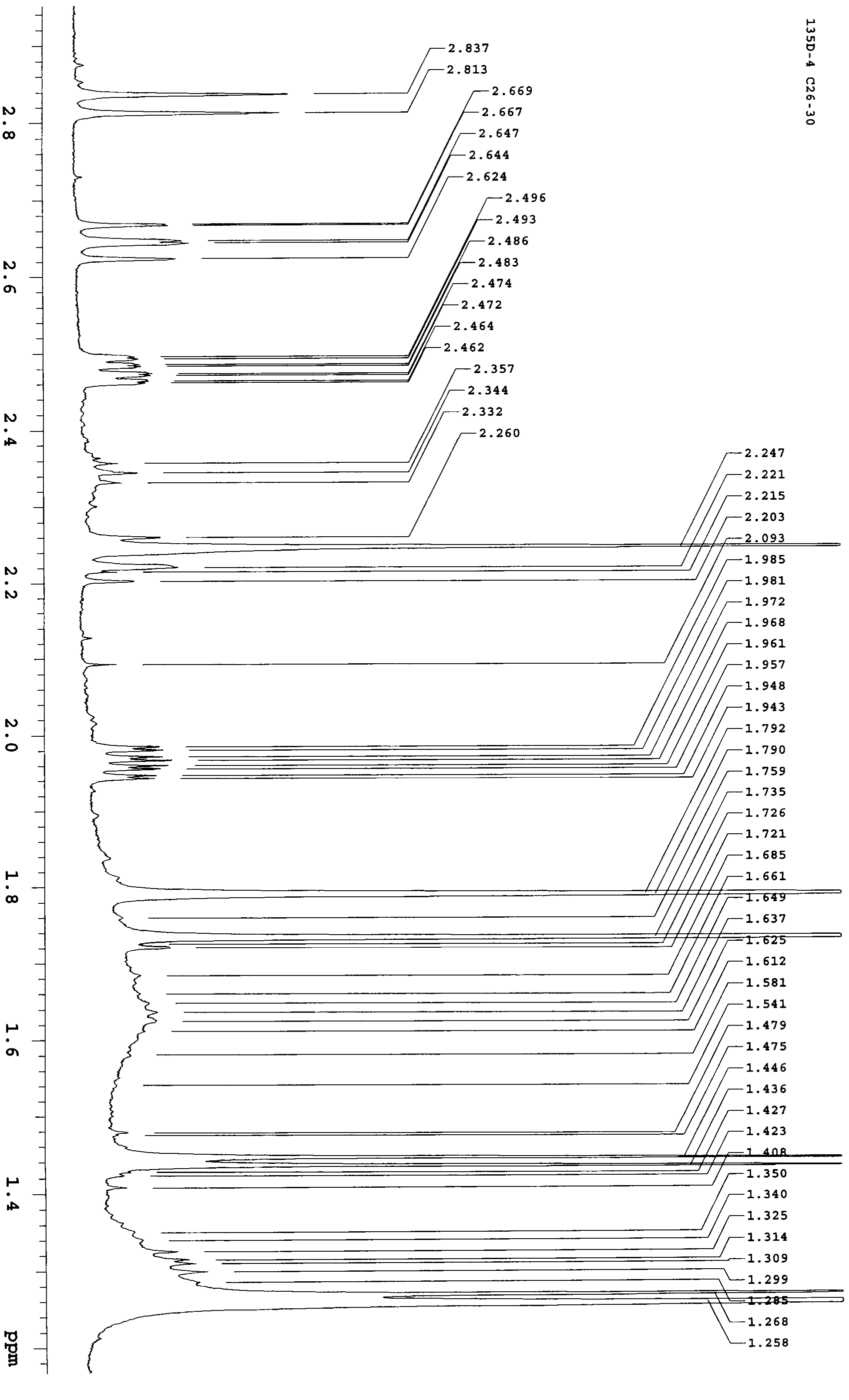
135D-4 C26-30
2.837
2.813
2.669
2.667
2.647
2.644
2.624
2.496
2.493
2.486
2.483
2.474
2.472
2.464
2.462
2.357
2.344
2.332
2.260
2.247
2.221
2.215
2.203
2.093
1.985
1.981
1.972
1.968
1.961
1.957
1.948
1.943
1.792
1.790
1.759
1.735
1.726
1.721
1.685
1.661
1.649
1.637
1.625
1.612
1.581
1.541
1.479
1.475
1.446
1.436
1.427
1.423
1.408
1.350
1.340
1.325
1.314
1.309
1.299
1.285
1.268
1.258
2.8
2.6
2.4
2.2
2.0
1.8
1.6
1.4
ppm

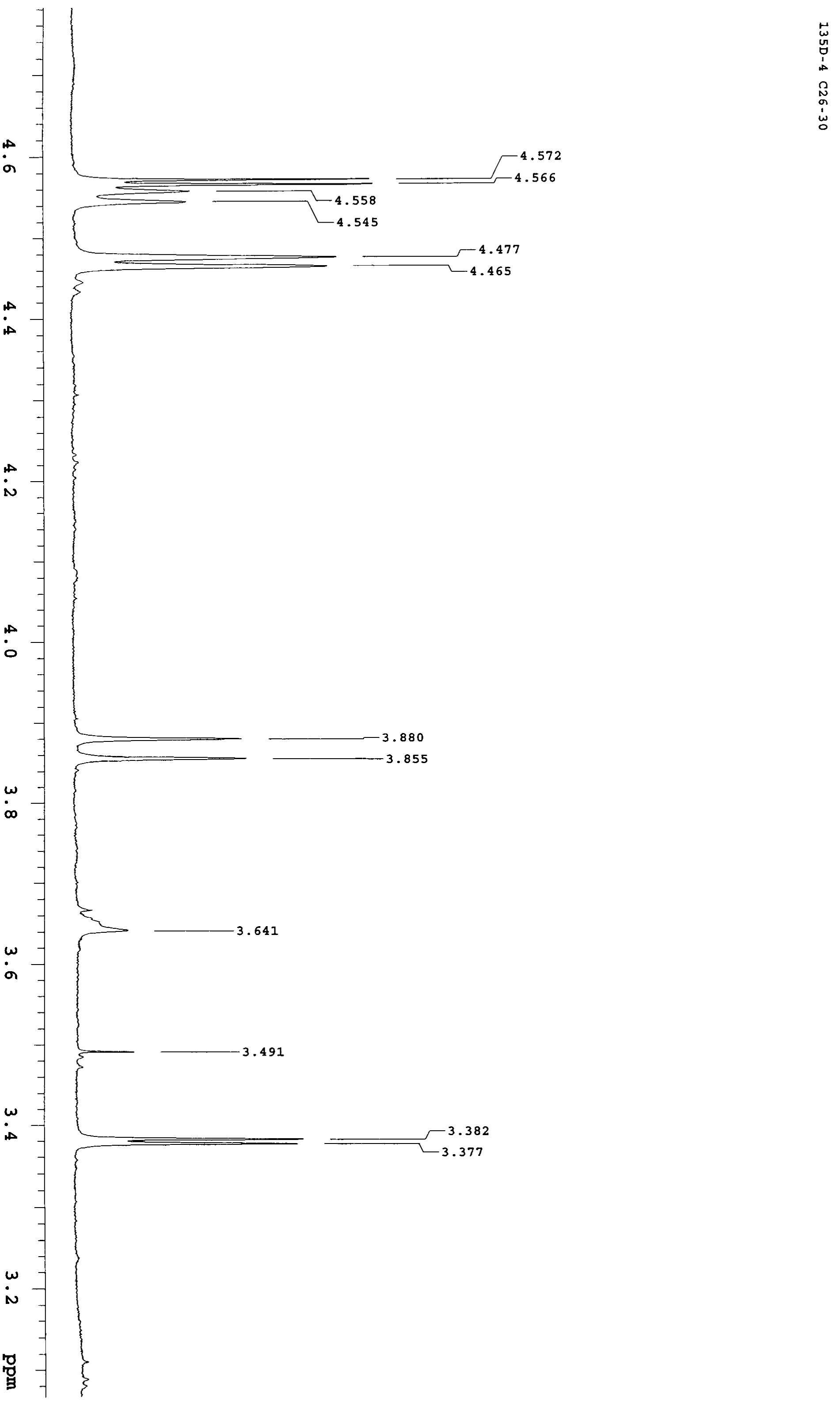
135D-4 C26-30
4.572
4.566
4.558
4.545
4.477
4.465
3.880
3.855
3.641
3.491
3.382
3.377
4.6
4.4
4.2
4.0
3.8
3.6
3.4
3.2
ppm

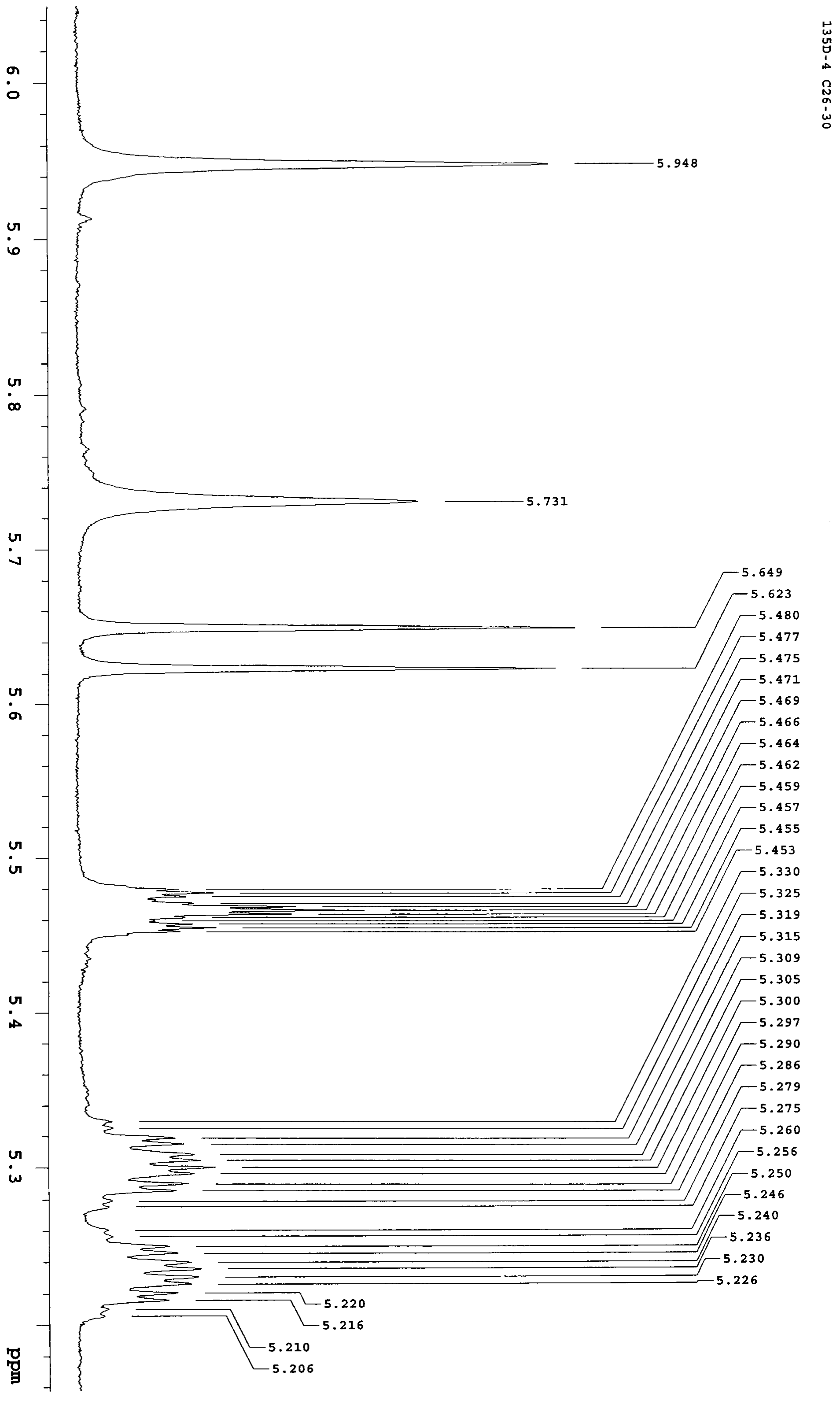
135D-4 C26-30
5.948
5.731
5.649
5.623
5.480
5.477
5.475
5.471
5.469
5.466
5.464
5.462
5.459
5.457
5.455
5.453
5.330
5.325
5.319
5.315
5.309
5.305
5.300
5.297
5.290
5.286
5.279
5.275
5.260
5.256
5.250
5.246
5.240
5.236
5.230
5.226
5.220
5.216
5.210
5.206
6.0
5.9
5.8
5.7
5.6
5.5
5.4
5.3
ppm

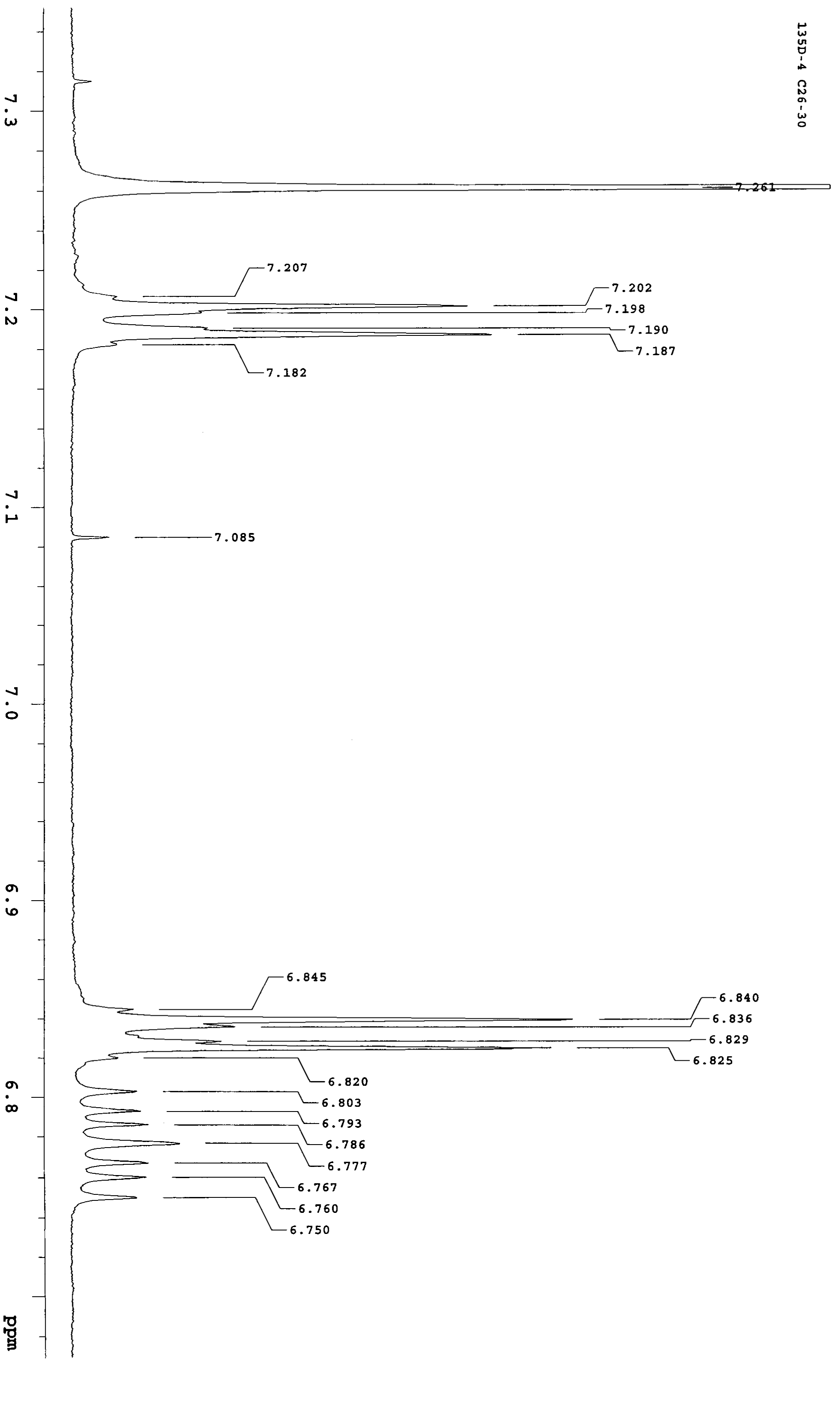
135D-4 C26-30
7.3
7.261
7.207
7.202
7.198
7.190
7.187
7.182
7.2
7.1
7.085
7.0
6.9
6.845
6.840
6.836
6.829
6.825
6.820
6.803
6.793
6.786
6.777
6.767
6.760
6.750
6.8
ppm

**Figure S3 $^{13}C$ NMR spectrum of 1 in $CDCl_3$**

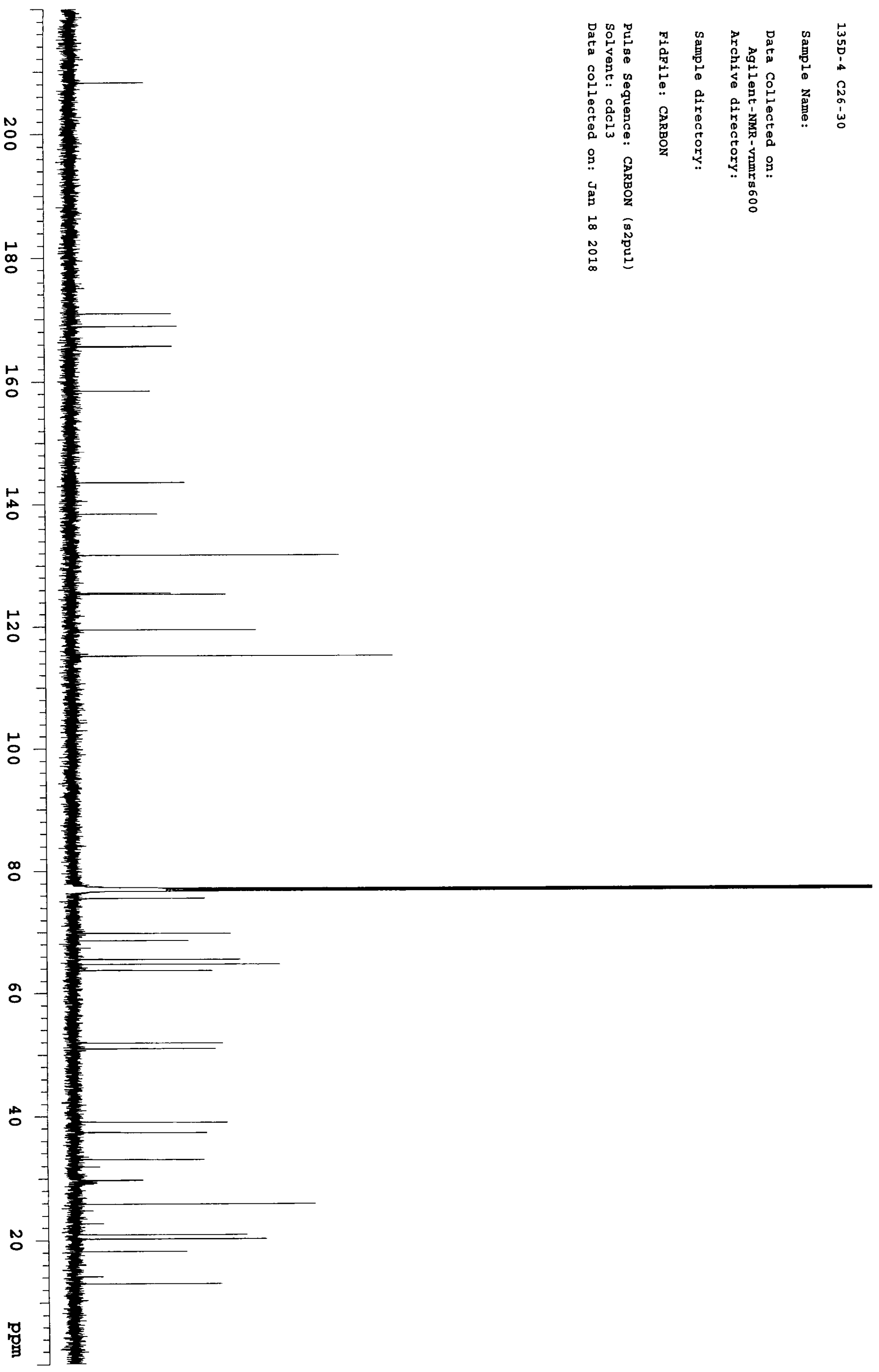

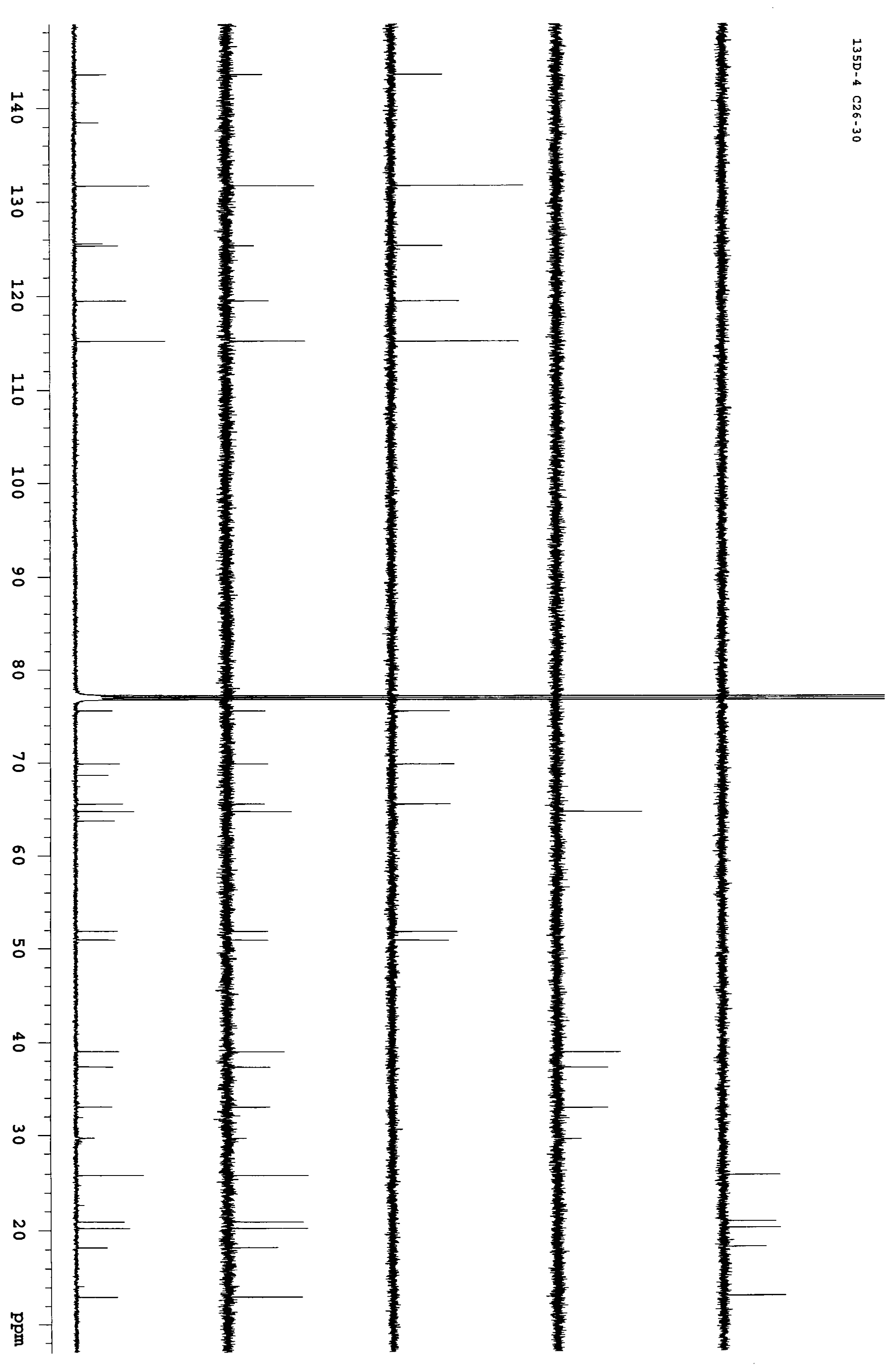
135D-4 C26-30
140
130
120
110
100
90
80
70
60
50
40
30
20
ppm

**Figure S4 $^1$H-$^1$H COSY of 1**

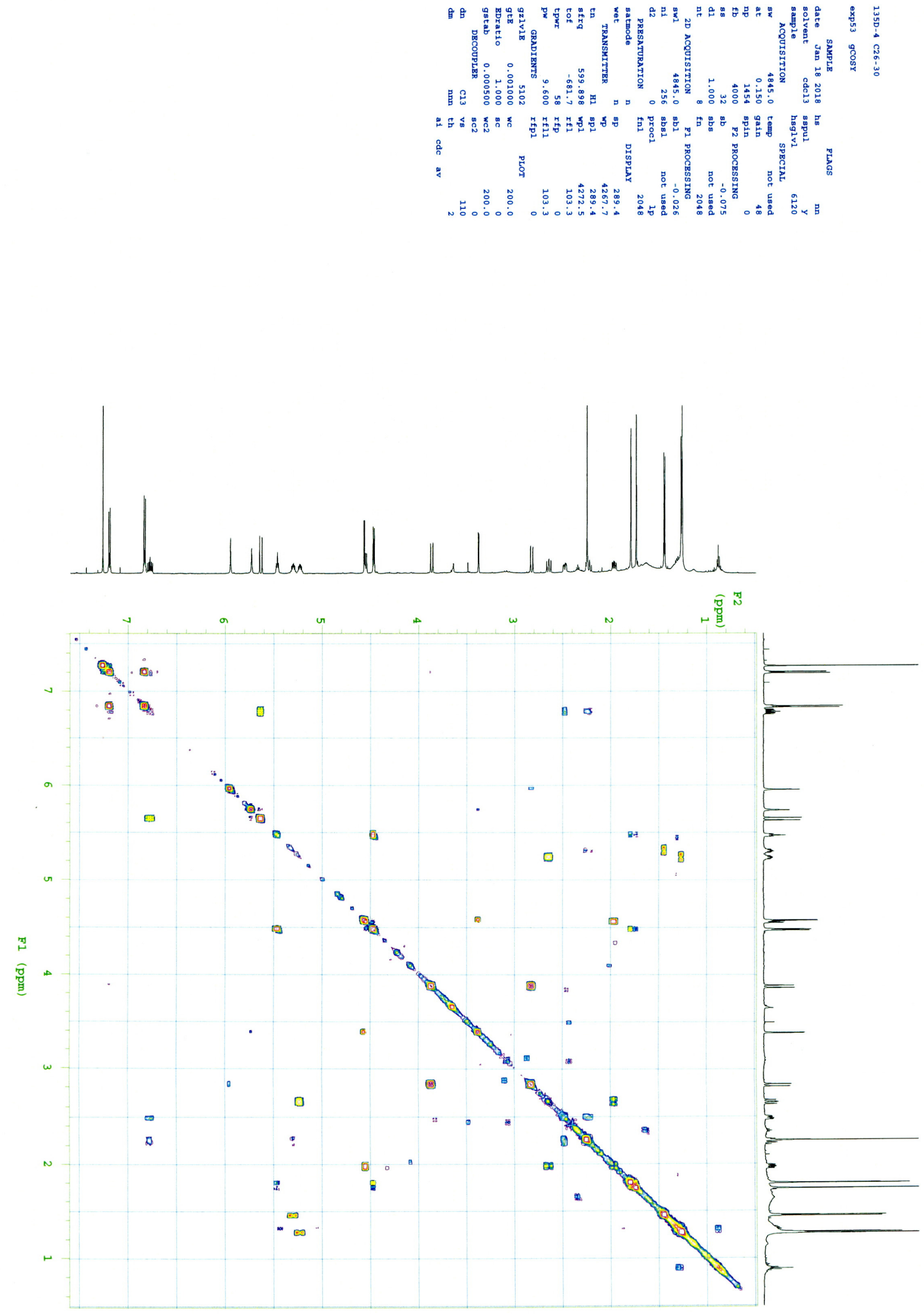

135D-4 C26-30

exp53 gCOSY

```
      SAMPLE                FLAGS
date    Jan 18 2018   hs              nn
solvent       cdcl3   sspul            y
sample                hsglvl        6120
   ACQUISITION          SPECIAL
sw           4845.0   temp      not used
at            0.150   gain            48
np             1454   spin             0
fb             4000     F2 PROCESSING
ss               32   sb          -0.075
d1            1.000   sbs       not used
nt                8   fn            2048
  2D ACQUISITION        F1 PROCESSING
sw1          4845.0   sb1         -0.026
ni              256   sbs1      not used
d2                0   proc1           lp
  PRESATURATION       fn1           2048
satmode           n        DISPLAY
wet               n   sp          2546.3
   TRANSMITTER        wp          1902.0
tn               H1   sp1          672.7
sfrq        599.898   wp1         3775.7
tof          -681.7   rfl          103.3
tpwr             58   rfp              0
pw            9.600   rfl1         103.3
    GRADIENTS         rfp1             0
gzlvlE         5102          PLOT
gtE        0.001000   wc           250.0
EDratio       1.000   sc               0
gstab      0.000500   wc2          200.0
    DECOUPLER         sc2              0
dn              C13   vs             110
dm              nnn   th               2
                      ai   cdc   av
```

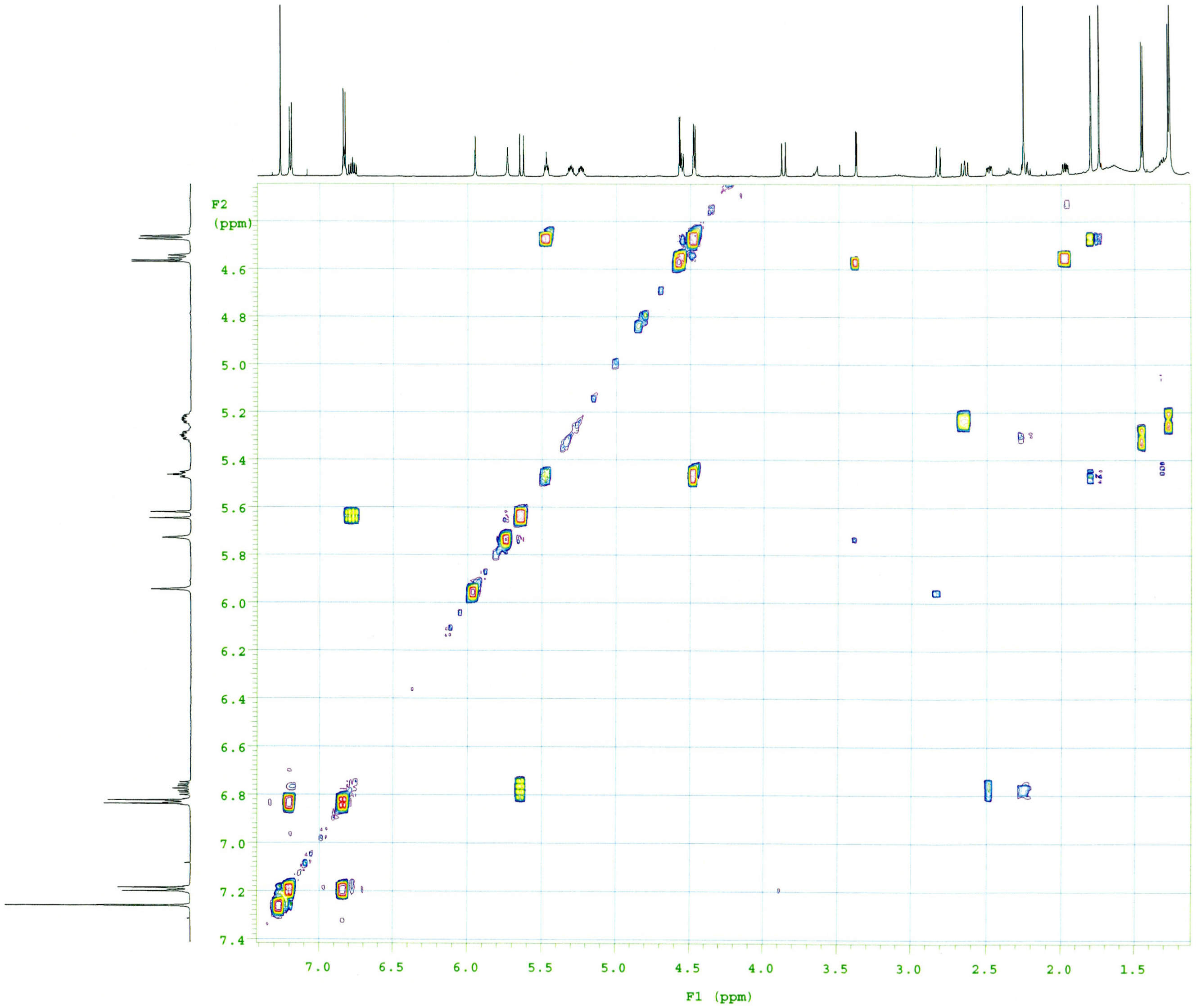

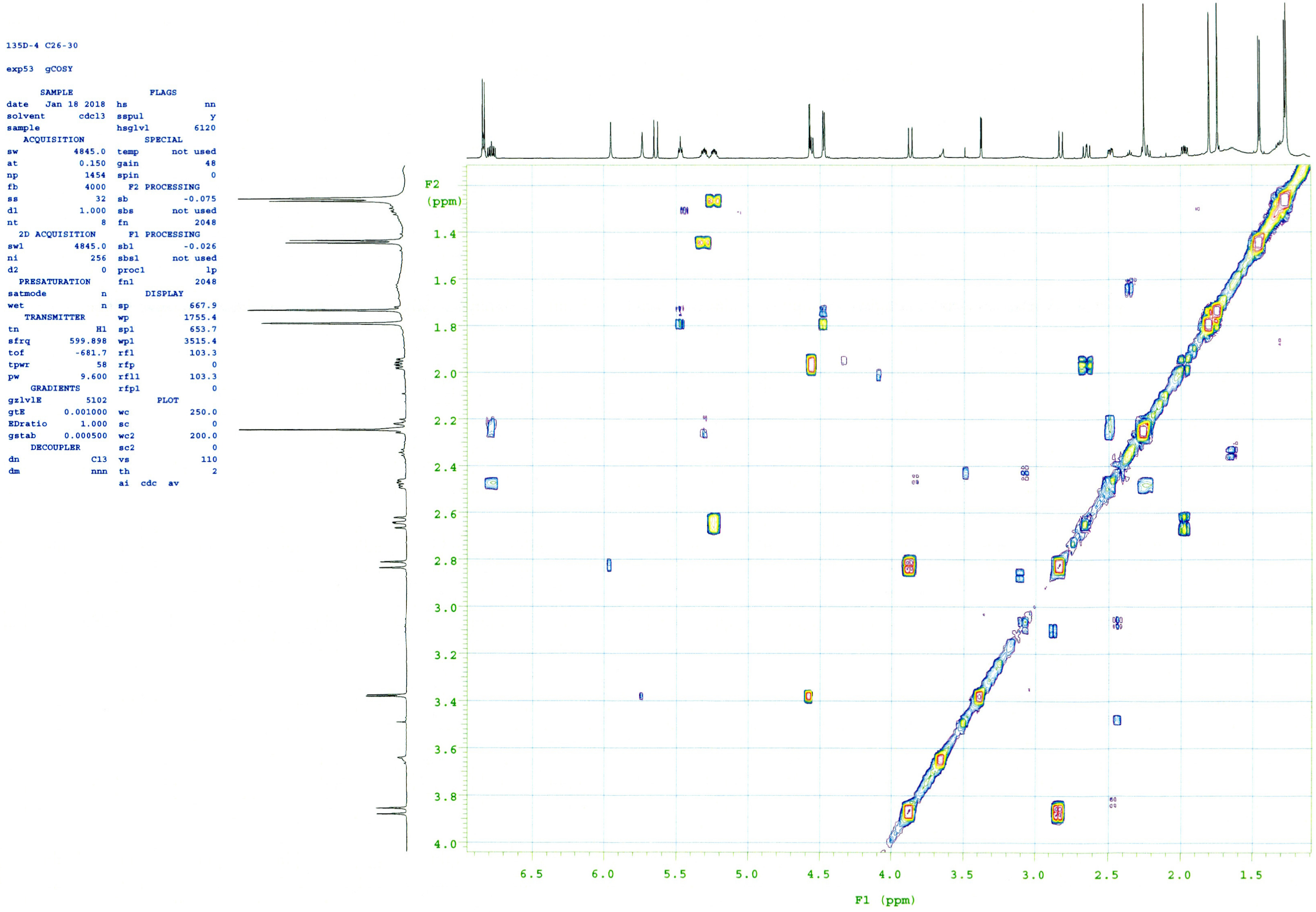
135D-4 C26-30
exp53 gCOSY
SAMPLE
date Jan 18 2018
solvent cdcl3
sample
ACQUISITION
sw 4845.0
at 0.150
np 1454
fb 4000
ss 32
d1 1.000
nt 8
2D ACQUISITION
sw1 4845.0
ni 256
d2 0
PRESATURATION
satmode n
wet n
TRANSMITTER
tn H1
sfrq 599.898
tof -681.7
tpwr 58
pw 9.600
GRADIENTS
gzlvlE 5102
gtE 0.001000
EDratio 1.000
gstab 0.000500
DECOUPLER
dn C13
dm nnn
FLAGS
hs nn
sspul y
hsglvl 6120
SPECIAL
temp not used
gain 48
spin 0
F2 PROCESSING
sb -0.075
sbs not used
fn 2048
F1 PROCESSING
sb1 -0.026
sbs1 not used
proc1 lp
fn1 2048
DISPLAY
sp 667.9
wp 1755.4
sp1 653.7
wp1 3515.4
rfl 103.3
rfp 0
rfl1 103.3
rfp1 0
PLOT
wc 250.0
sc 0
wc2 200.0
sc2 0
vs 110
th 2
ai cdc av
F2 (ppm)
F1 (ppm)

**Figure S5 NOESY of 1**

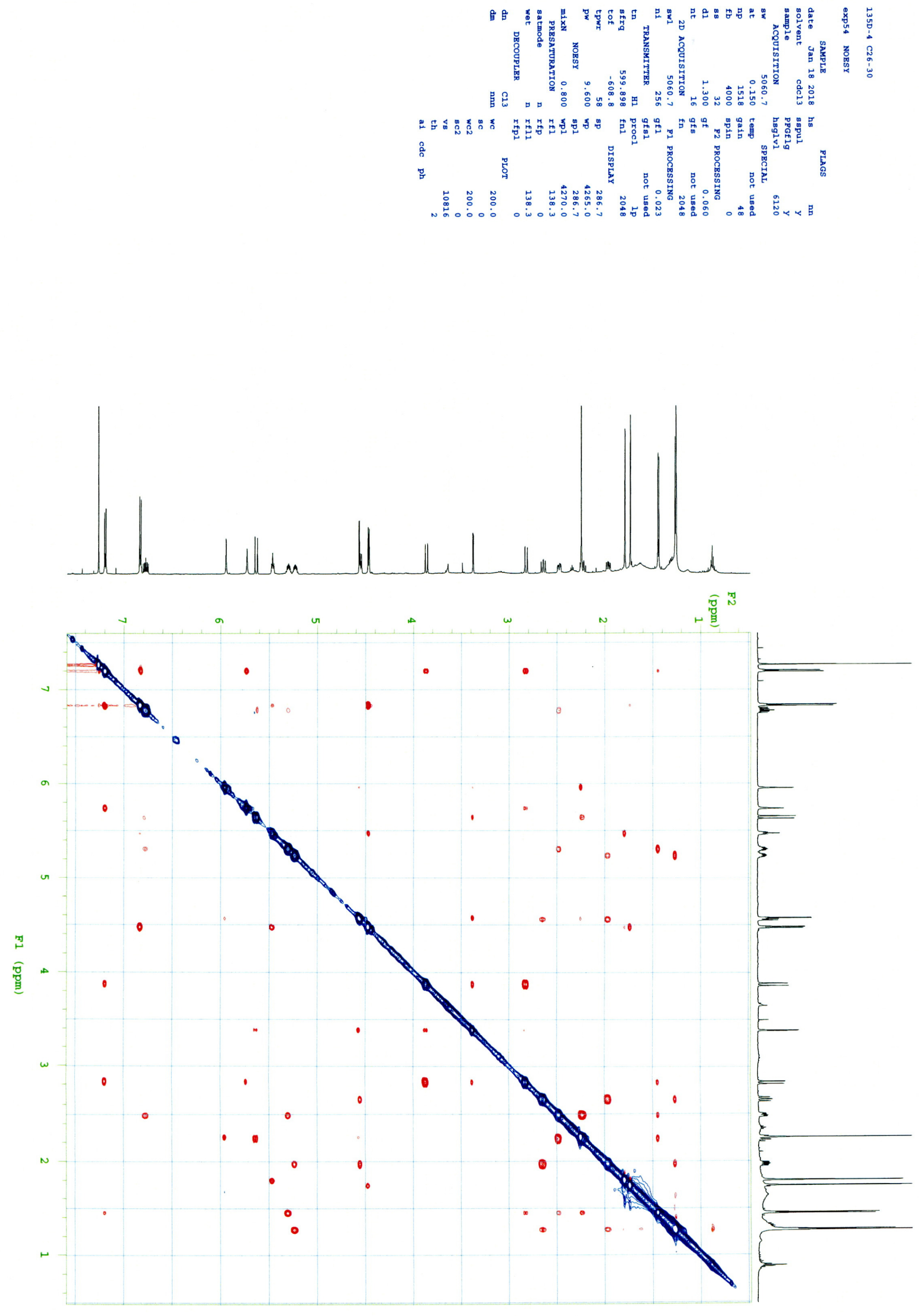

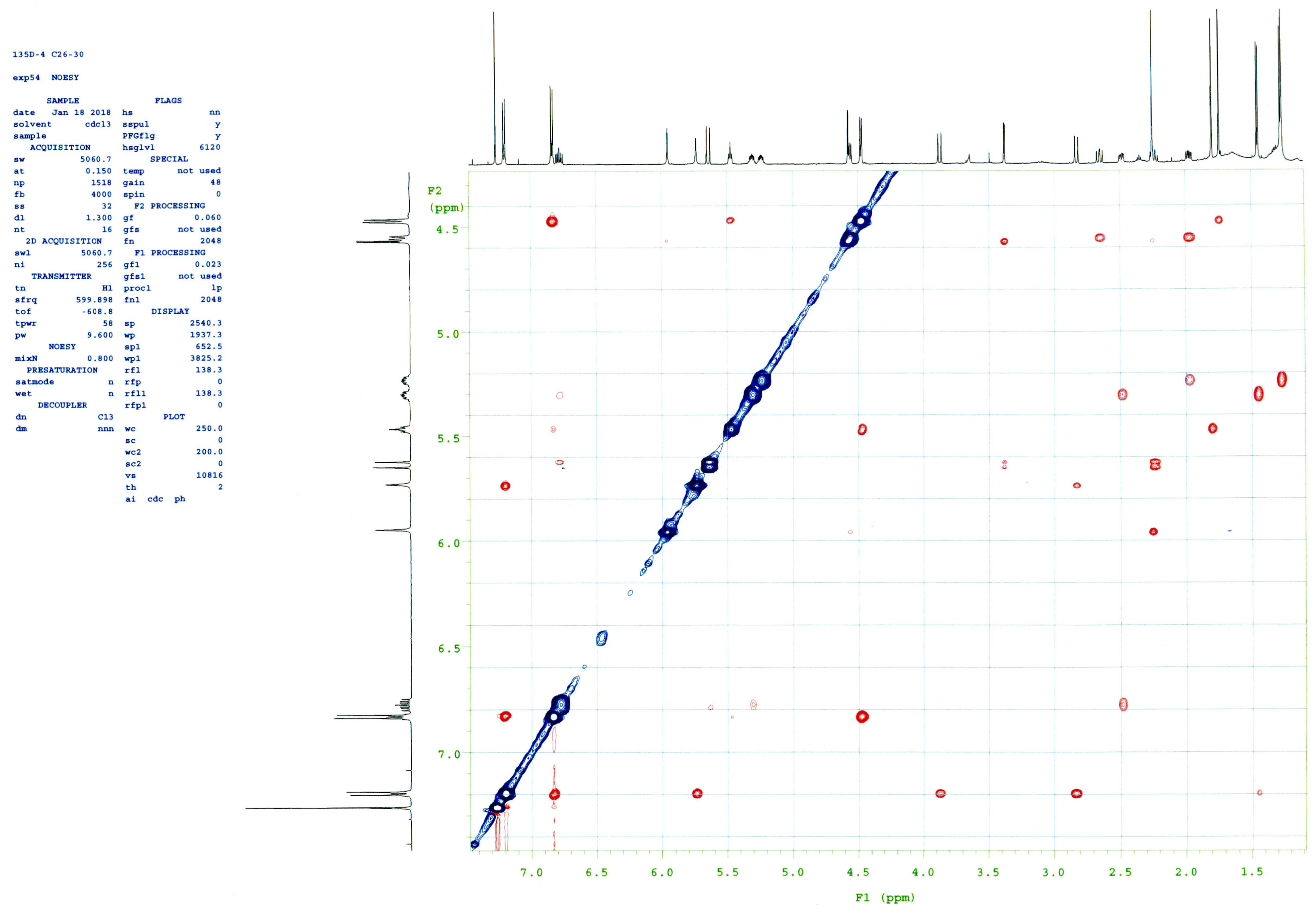

135D-4 C26-30
exp54 NOESY
SAMPLE
date Jan 18 2018
solvent cdcl3
sample
ACQUISITION
sw 5060.7
at 0.150
np 1518
fb 4000
ss 32
d1 1.300
nt 16
2D ACQUISITION
sw1 5060.7
ni 256
TRANSMITTER
tn H1
sfrq 599.898
tof -608.8
tpwr 58
pw 9.600
NOESY
mixN 0.800
PRESATURATION
satmode n
wet n
DECOUPLER
dn C13
dm nnn
FLAGS
hs nn
sspul y
PFGflg y
hsglvl 6120
SPECIAL
temp not used
gain 48
spin 0
F2 PROCESSING
gf 0.060
gfs not used
fn 2048
F1 PROCESSING
gf1 0.023
gfs1 not used
proc1 lp
fn1 2048
DISPLAY
sp 2540.3
wp 1937.3
sp1 652.5
wp1 3825.2
rfl 138.3
rfp 0
rfl1 138.3
rfp1 0
PLOT
wc 250.0
sc 0
wc2 200.0
sc2 0
vs 10816
th 2
ai cdc ph
F2 (ppm)
4.5
5.0
5.5
6.0
6.5
7.0
F1 (ppm)
7.0
6.5
6.0
5.5
5.0
4.5
4.0
3.5
3.0
2.5
2.0
1.5

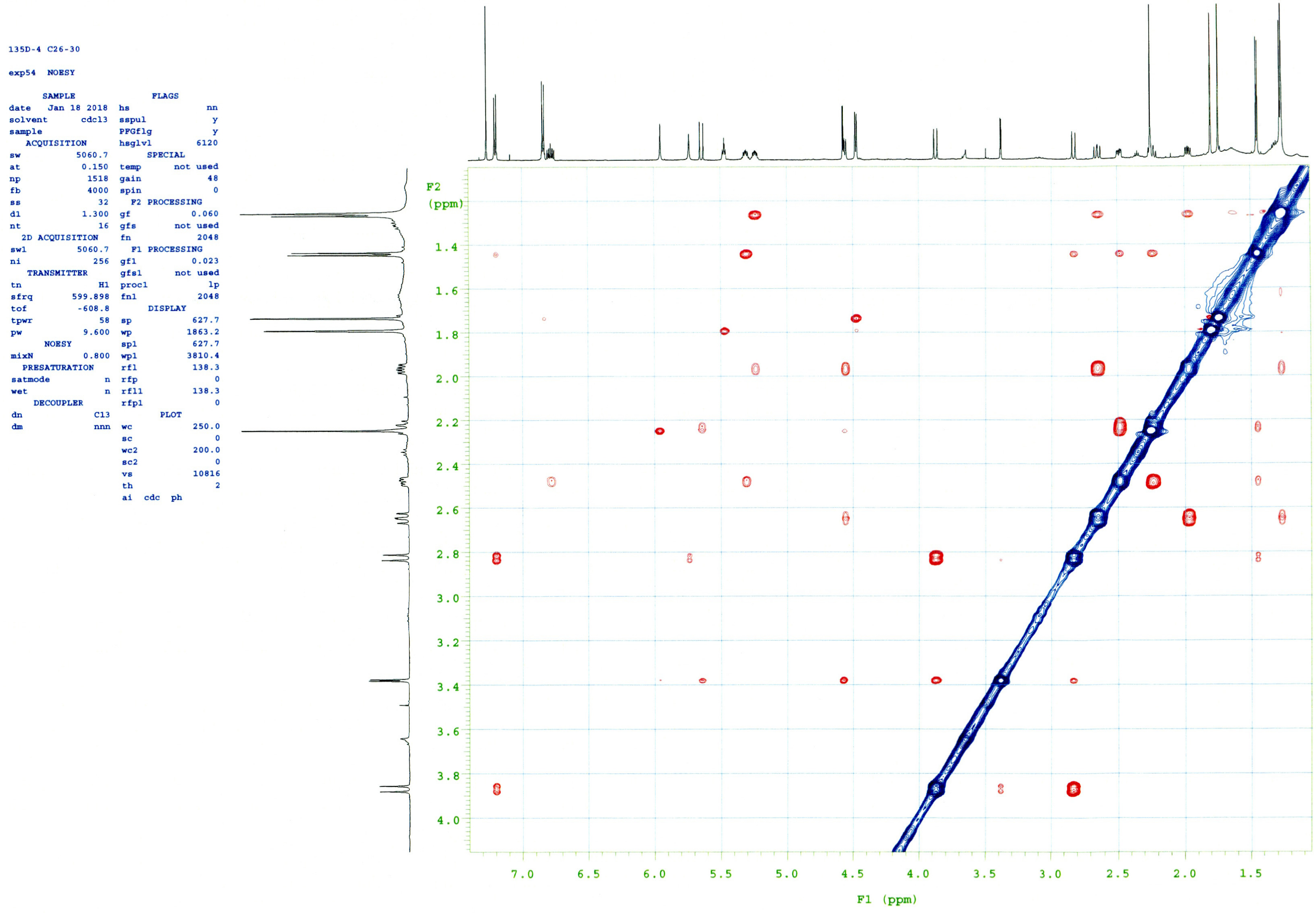
135D-4 C26-30
exp54 NOESY
SAMPLE
date Jan 18 2018
solvent cdcl3
sample
ACQUISITION
sw 5060.7
at 0.150
np 1518
fb 4000
ss 32
d1 1.300
nt 16
2D ACQUISITION
sw1 5060.7
ni 256
TRANSMITTER
tn H1
sfrq 599.898
tof -608.8
tpwr 58
pw 9.600
NOESY
mixN 0.800
PRESATURATION
satmode n
wet n
DECOUPLER
dn C13
dm nnn
FLAGS
hs nn
sspul y
PFGflg y
hsglvl 6120
SPECIAL
temp not used
gain 48
spin 0
F2 PROCESSING
gf 0.060
gfs not used
fn 2048
F1 PROCESSING
gf1 0.023
gfs1 not used
proc1 lp
fn1 2048
DISPLAY
sp 627.7
wp 1863.2
sp1 627.7
wp1 3810.4
rfl 138.3
rfp 0
rfl1 138.3
rfp1 0
PLOT
wc 250.0
sc 0
wc2 200.0
sc2 0
vs 10816
th 2
ai cdc ph
F2 (ppm)
1.4
1.6
1.8
2.0
2.2
2.4
2.6
2.8
3.0
3.2
3.4
3.6
3.8
4.0
7.0
6.5
6.0
5.5
5.0
4.5
4.0
3.5
3.0
2.5
2.0
1.5
F1 (ppm)

## Figure S6 HMQC of 1

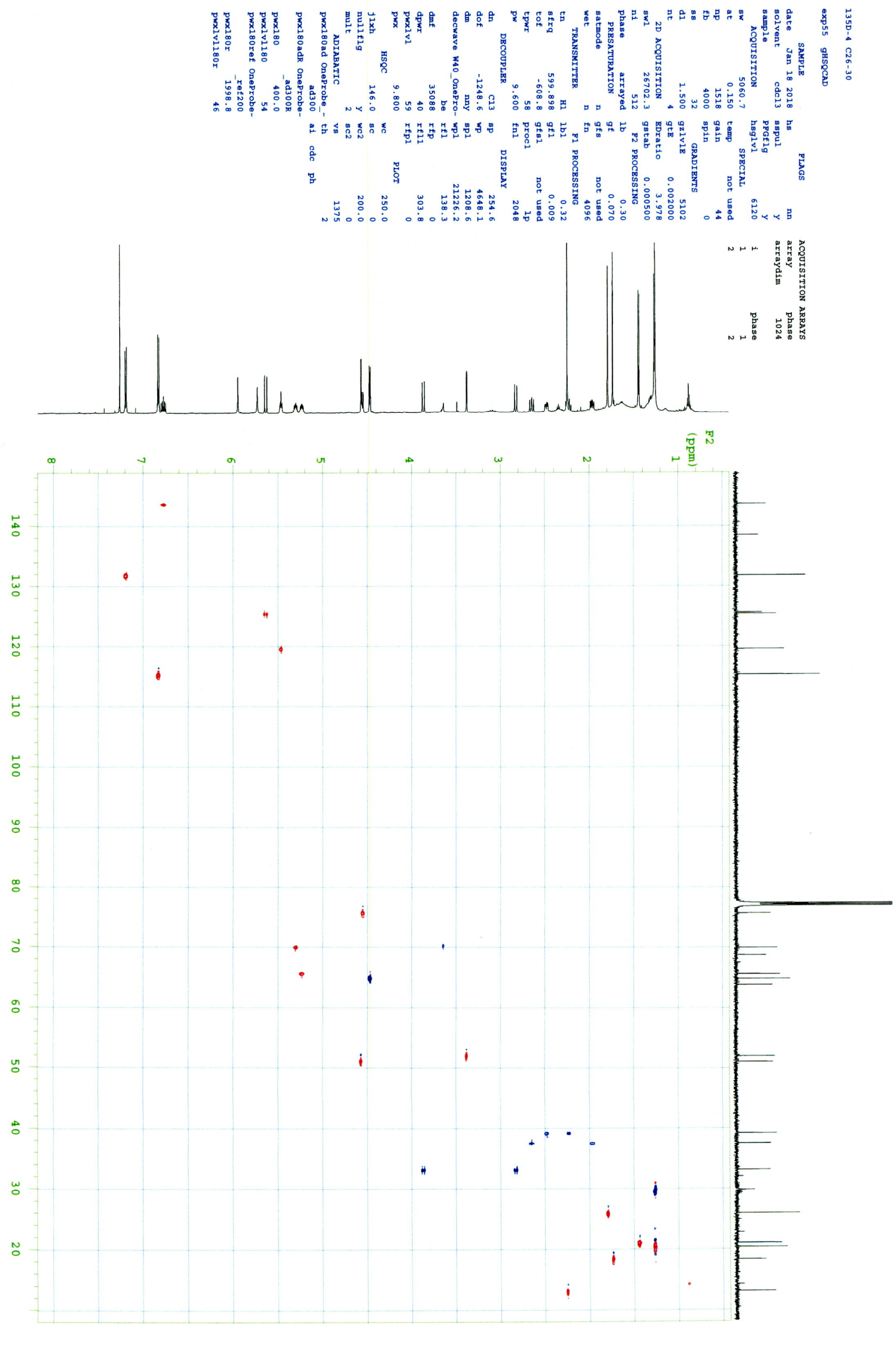

135D-4 C26-30

exp55 gHSQCAD

```
      SAMPLE                FLAGS            ACQUISITION ARRAYS
date   Jan 18 2018   hs                nn    array          phase
solvent       cdcl3  sspul              y    arraydim        1024
sample               PFGflg             y
   ACQUISITION       hsglvl          6120     i             phase
sw           5060.7         SPECIAL           1                 1
at            0.150  temp        not used     2                 2
np             1518  gain              44
fb             4000  spin               0
ss               32        GRADIENTS
d1            1.500  gzlvlE          5102
nt                4  gtE         0.002000
  2D ACQUISITION     EDratio        3.978
sw1         26702.3  gstab       0.000500
ni              512    F2 PROCESSING
phase       arrayed  lb              0.30
  PRESATURATION      gf             0.070
satmode           n  gfs         not used
wet               n  fn              4096
   TRANSMITTER         F1 PROCESSING
tn               H1  lb1             0.32
sfrq        599.898  gf1            0.009
tof          -608.8  gfs1        not used
tpwr             58  proc1             lp
pw            9.600  fn1             2048
     DECOUPLER             DISPLAY
dn              C13  sp            3044.4
dof         -1248.6  wp            1596.3
dm              nny  sp1          16906.6
decwave W40_OnePro-  wp1           4902.4
                 be  rf1            138.3
dmf           35088  rfp                0
dpwr             40  rf11           303.8
pwxlvl           59  rfp1               0
pwx           9.800         PLOT
        HSQC         wc             250.0
j1xh          146.0  sc                 0
nullflg           y  wc2            200.0
mult              2  sc2                0
     ADIABATIC       vs              1375
pwx180ad OneProbe_-  th                 2
              ad300  ai  cdc  ph
pwx180adR OneProbe-
             _ad300R
pwx180        400.0
pwxlvl180        54
pwx180ref OneProbe-
             _ref200
pwx180r      1998.8
pwxlvl180r       46
```

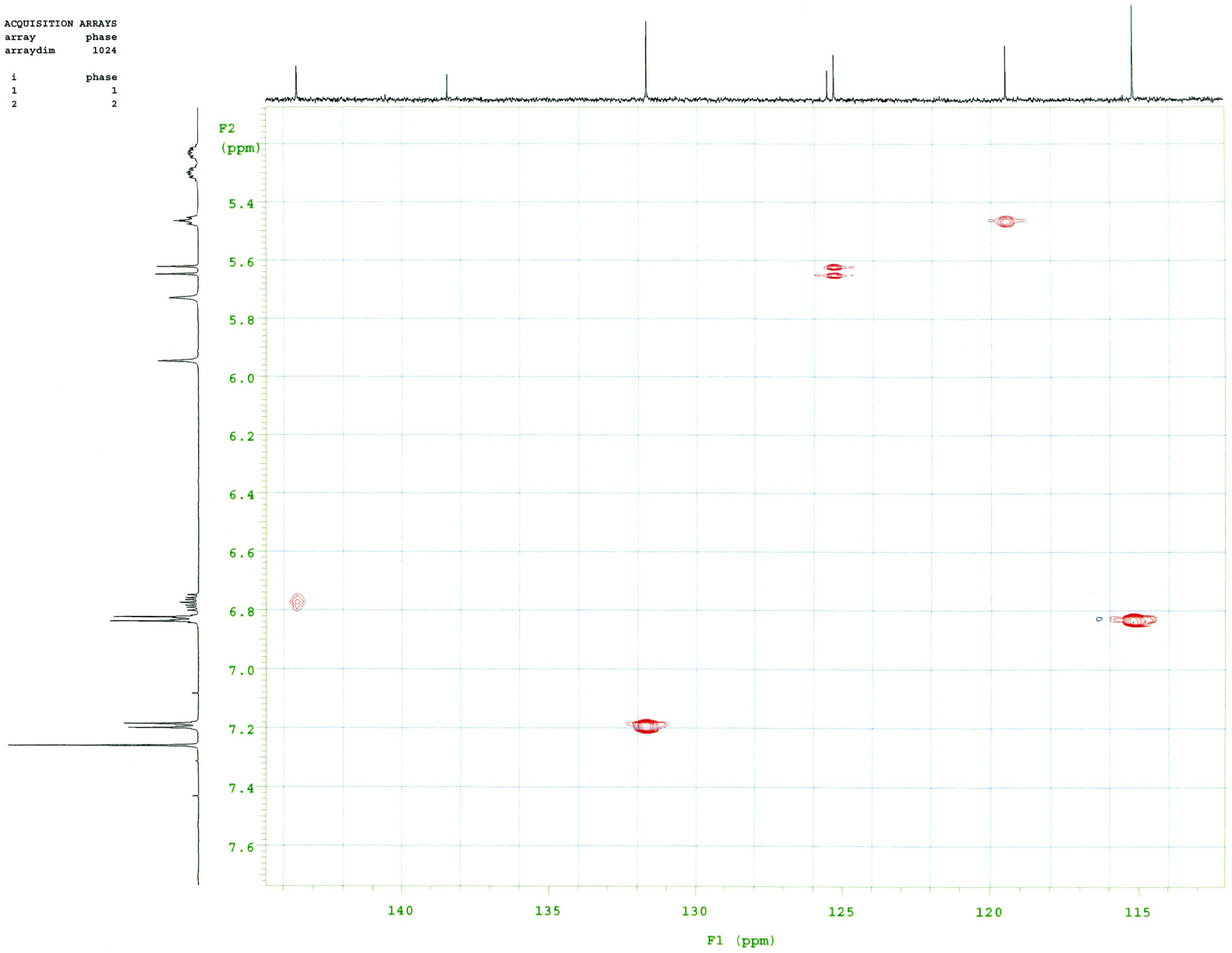

135D-4 C26-30

exp55 gHSQCAD

```
      SAMPLE                 FLAGS              ACQUISITION ARRAYS
date    Jan 18 2018   hs                 nn    array          phase
solvent       cdcl3   sspul               y    arraydim        1024
sample                PFGflg              y
   ACQUISITION        hsglvl           6120     i             phase
sw           5060.7          SPECIAL            1                 1
at            0.150   temp         not used     2                 2
np             1518   gain               44
fb             4000   spin                0
ss               32         GRADIENTS
d1            1.500   gzlvlE           5102
nt                4   gtE          0.002000
  2D ACQUISITION      EDratio         3.978
sw1         26702.3   gstab        0.000500
ni              512     F2 PROCESSING
phase       arrayed   lb               0.30
  PRESATURATION       gf              0.070
satmode           n   gfs          not used
wet               n   fn               4096
   TRANSMITTER           F1 PROCESSING
tn               H1   lb1              0.32
sfrq        599.898   gfl             0.009
tof          -608.8   gfs1         not used
tpwr             58   procl              lp
pw            9.600   fn1              2048
    DECOUPLER              DISPLAY
dn              C13   sp             1932.5
dof         -1248.6   wp             1386.3
dm              nny   sp1            4546.4
decwave W40_OnePro~   wp1            7510.0
                 be   rfl             138.3
dmf           35088   rfp                 0
dpwr             40   rfl1            303.8
pwxlvl           59   rfp1                0
pwx           9.800          PLOT
       HSQC           wc              250.0
j1xh          146.0   sc                  0
nullflg           y   wc2             200.0
mult              2   sc2                 0
     ADIABATIC        vs               1375
pwx180ad OneProbe_~   th                  2
              ad300   ai   cdc   ph
pwx180adR OneProbe~
             _ad300R
pwx180        400.0
pwxlvl180        54
pwx180ref OneProbe~
             _ref200
pwx180r      1998.8
pwxlvl180r       46
```

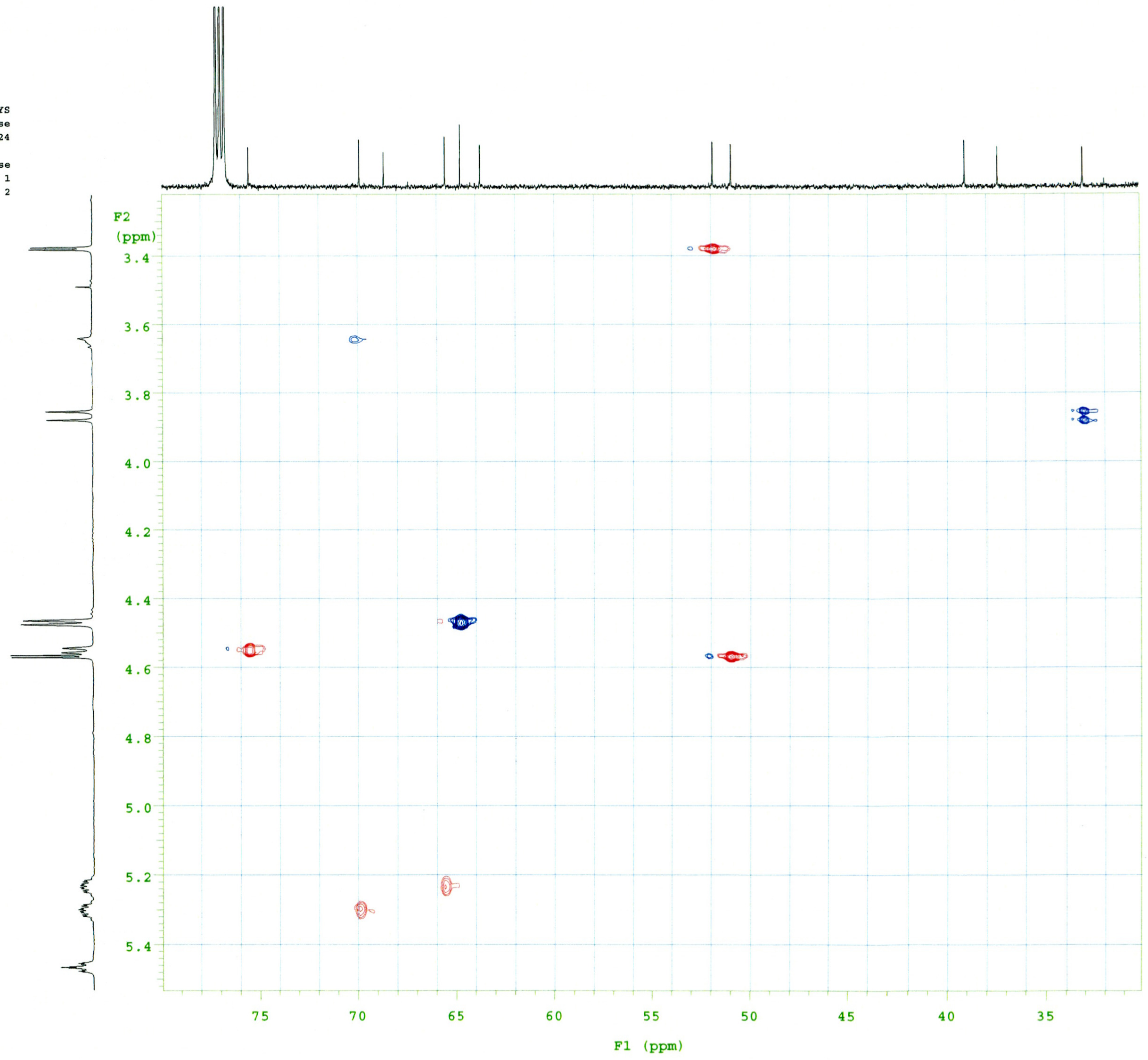

```
135D-4 C26-30

exp55  gHSQCAD

      SAMPLE                FLAGS              ACQUISITION ARRAYS
date    Jan 18 2018  hs                nn      array          phase
solvent       cdcl3  sspul              y      arraydim        1024
sample               PFGflg             y
    ACQUISITION      hsglvl          6120      i              phase
sw           5060.7         SPECIAL            1                  1
at            0.150  temp        not used      2                  2
np             1518  gain              44
fb             4000  spin               0
ss               32        GRADIENTS
d1            1.500  gzlvlE          5102
nt                4  gtE         0.002000
   2D ACQUISITION    EDratio        3.978
sw1         26702.3  gstab       0.000500
ni              512     F2 PROCESSING
phase       arrayed  lb              0.30
  PRESATURATION      gf             0.070
satmode           n  gfs         not used
wet               n  fn              4096
    TRANSMITTER         F1 PROCESSING
tn               H1  lb1             0.32
sfrq        599.898  gf1            0.009
tof          -608.8  gfs1        not used
tpwr             58  proc1             lp
pw            9.600  fn1             2048
     DECOUPLER            DISPLAY
dn              C13  sp             536.3
dof         -1248.6  wp            1944.7
dm              nny  sp1           1704.0
decwave W40_OnePro~  wp1           4354.8
                 be  rfl            138.3
dmf           35088  rfp                0
dpwr             40  rfl1           303.8
pwxlvl           59  rfp1               0
pwx           9.800          PLOT
        HSQC         wc             250.0
j1xh          146.0  sc                 0
nullflg           y  wc2            200.0
mult              2  sc2                0
     ADIABATIC       vs              1375
pwx180ad OneProbe_~  th                 2
              ad300  ai   cdc   ph
pwx180adR OneProbe~
             _ad300R
pwx180        400.0
pwxlvl180        54
pwx180ref OneProbe~
             _ref200
pwx180r      1998.8
pwxlvl180r       46
```

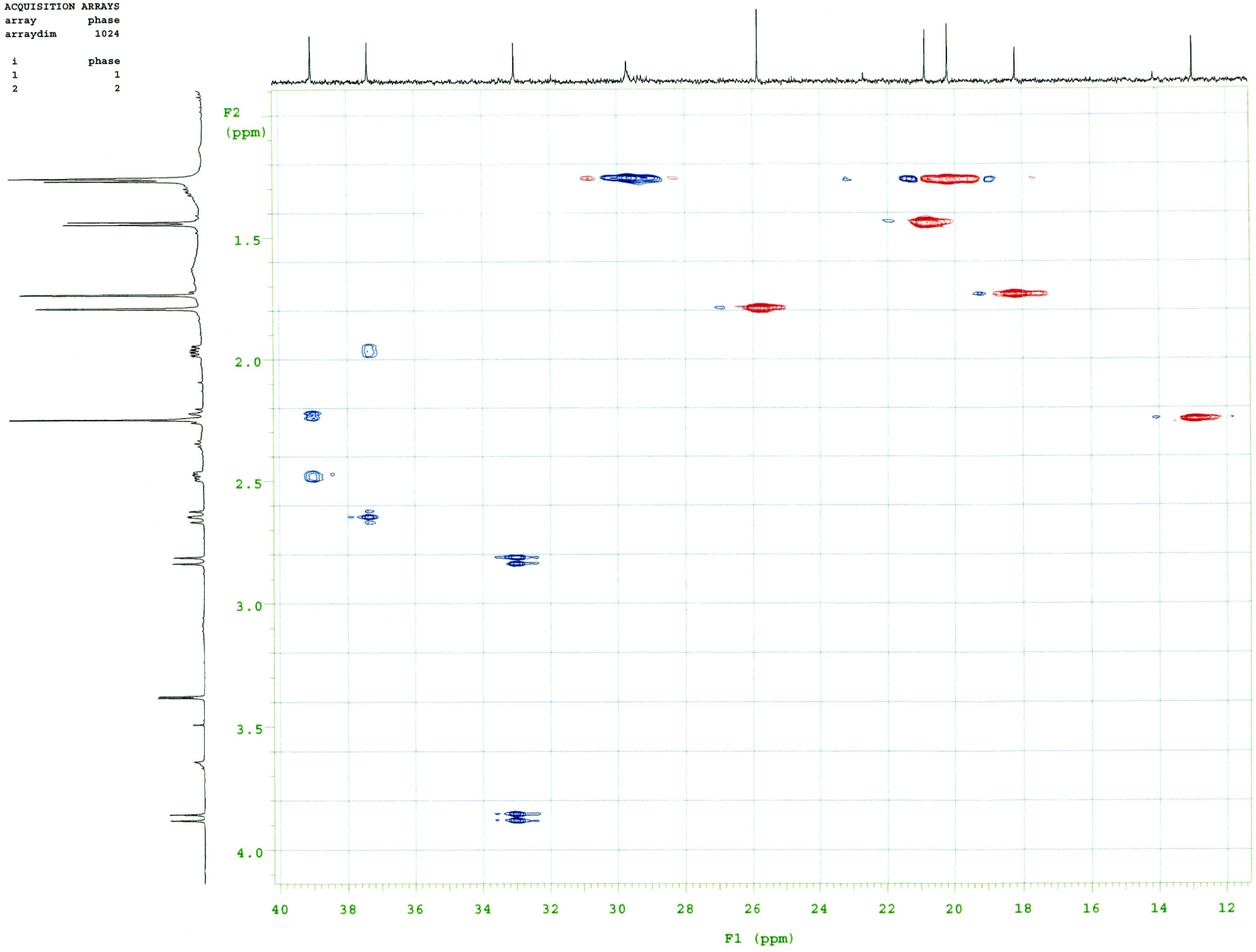

## Figure S7 HMBC of 1

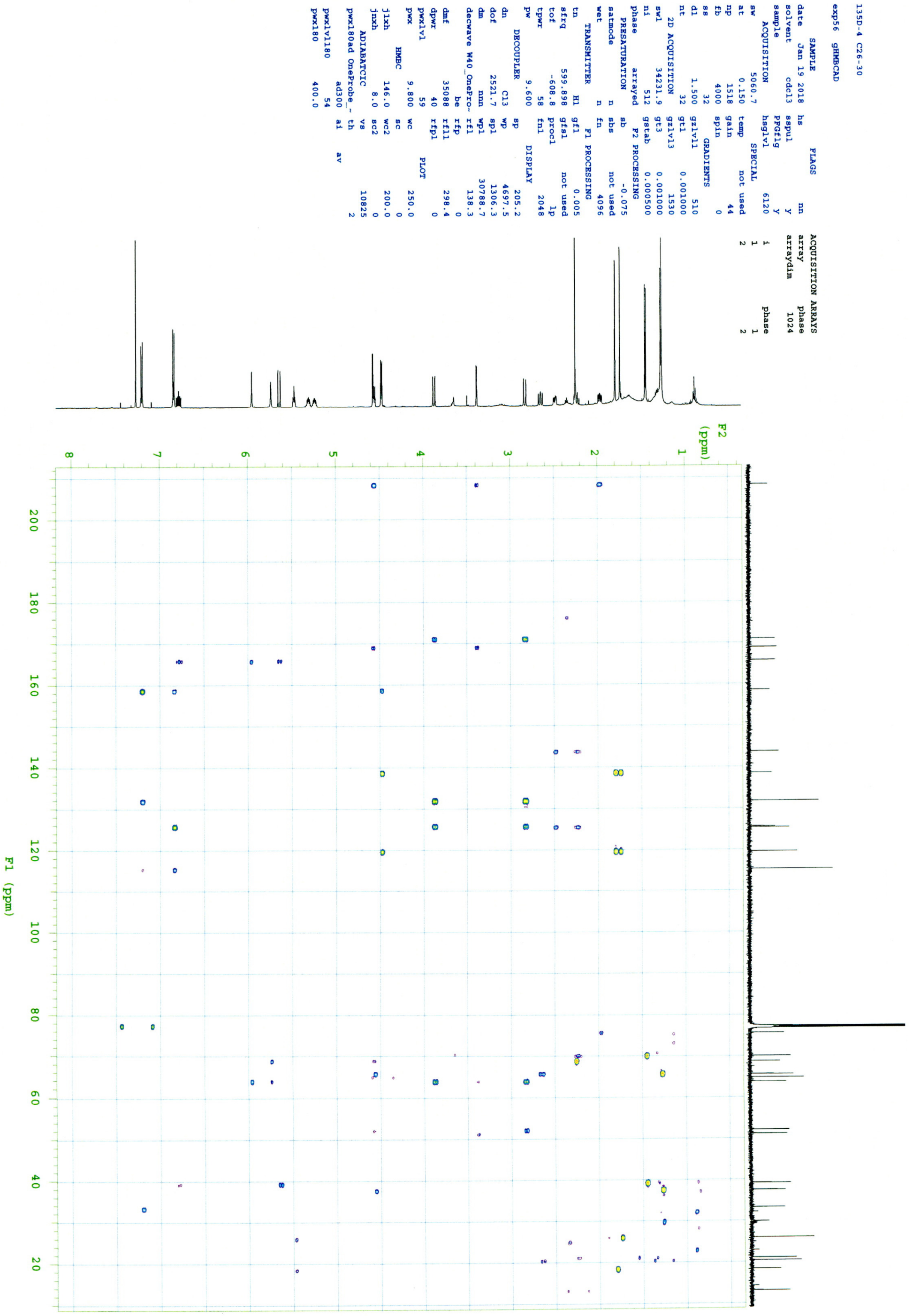

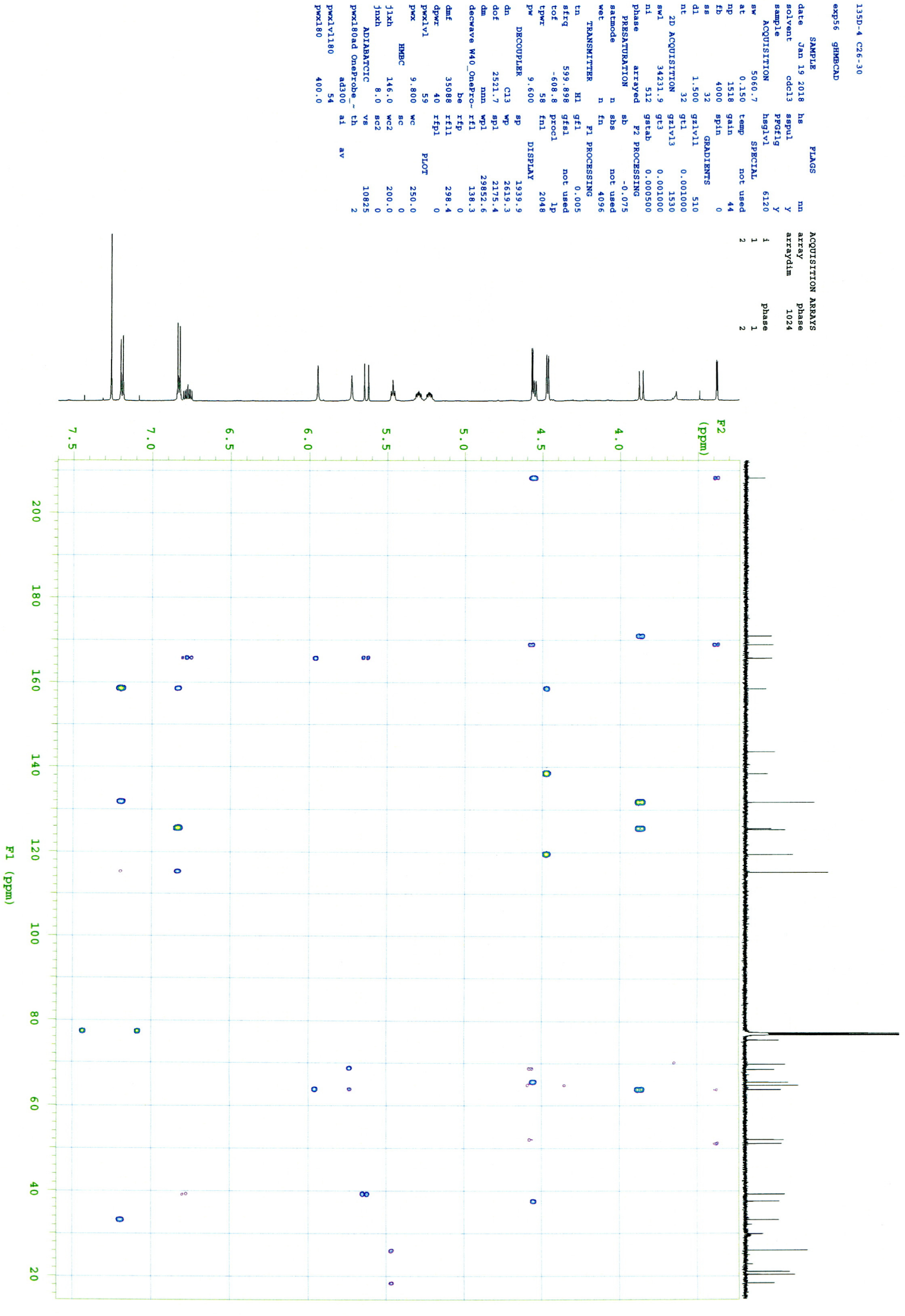
135D-4 C26-30
exp56 gHMBCAD
SAMPLE
date Jan 19 2018
solvent cdcl3
sample
ACQUISITION
sw 5060.7
at 0.150
np 1518
fb 4000
ss 32
d1 1.500
nt 32
2D ACQUISITION
sw1 34231.9
ni 512
phase arrayed
PRESATURATION
satmode n
wet n
TRANSMITTER
tn H1
sfrq 599.898
tof -608.8
tpwr 58
pw 9.600
DECOUPLER
dn C13
dof 2521.7
dm nnn
decwave W40_OnePro~ be
dmf 35088
dpwr 40
pwxlvl 59
pwx 9.800
HMBC
j1xh 146.0
jnxh 8.0
ADIABATCIC
pwx180ad OneProbe_~ ad300
pwxlvl180 54
pwx180 400.0
FLAGS
hs nn
sspul y
PFGflg y
hsglvl 6120
SPECIAL
temp not used
gain 44
spin 0
GRADIENTS
gzlvl1 510
gt1 0.001000
gzlvl3 1530
gt3 0.001000
gstab 0.000500
F2 PROCESSING
sb -0.075
sbs not used
fn 4096
F1 PROCESSING
gf1 0.005
gfs1 not used
proc1 lp
fn1 2048
DISPLAY
sp 1939.9
wp 2619.3
sp1 2175.4
wp1 29852.6
rfl 138.3
rfp 0
rfl1 298.4
rfp1 0
PLOT
wc 250.0
sc 0
wc2 200.0
sc2 0
vs 10825
th 2
ai av
ACQUISITION ARRAYS
array phase
arraydim 1024
i phase
1 1
2 2
F2 (ppm)
4.0
4.5
5.0
5.5
6.0
6.5
7.0
7.5
200
180
160
140
120
100
80
60
40
20
F1 (ppm)

```
135D-4 C26-30

exp56  gHMBCAD

      SAMPLE               FLAGS          ACQUISITION ARRAYS
date    Jan 19 2018  hs               nn  array          phase
solvent       cdcl3  sspul             y  arraydim        1024
sample               PFGflg            y
   ACQUISITION       hsglvl         6120   i             phase
sw           5060.7       SPECIAL          1                 1
at            0.150  temp       not used   2                 2
np             1518  gain             44
fb             4000  spin              0
ss               32      GRADIENTS
d1            1.500  gzlvl1          510
nt               32  gt1        0.001000
  2D ACQUISITION     gzlvl3         1530
sw1         34231.9  gt3        0.001000
ni              512  gstab      0.000500
phase       arrayed     F2 PROCESSING
  PRESATURATION      sb           -0.075
satmode           n  sbs        not used
wet               n  fn             4096
   TRANSMITTER          F1 PROCESSING
tn               H1  gf1           0.005
sfrq        599.898  gfs1       not used
tof          -608.8  proc1            lp
tpwr             58  fn1            2048
pw            9.600        DISPLAY
    DECOUPLER        sp            635.2
dn              C13  wp           1228.1
dof          2521.7  sp1          1506.8
dm              nnn  wp1         30521.2
decwave W40_OnePro-  rf1           138.3
                 be  rfp               0
dmf           35088  rfl1          298.4
dpwr             40  rfp1              0
pwxlvl           59        PLOT
pwx           9.800  wc            250.0
       HMBC          sc                0
j1xh          146.0  wc2           200.0
jnxh            8.0  sc2               0
    ADIABATCIC       vs            10825
pwx180ad OneProbe_~  th                2
              ad300  ai    av
pwxlvl180        54
pwx180        400.0
```

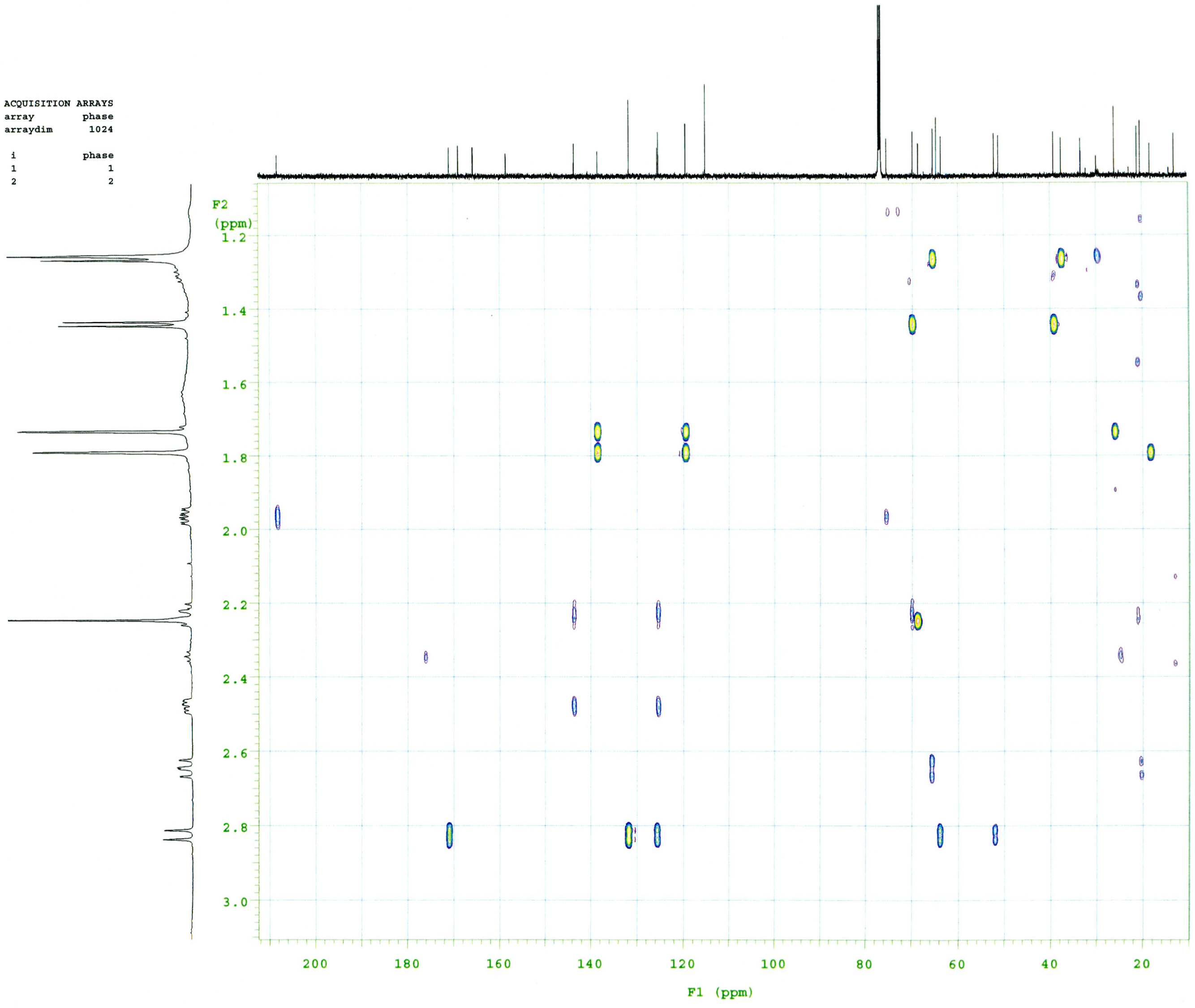

135D-4 C26-30

exp56 gHMBCAD

| SAMPLE | | FLAGS | | ACQUISITION ARRAYS | |
|---|---|---|---|---|---|
| date | Jan 19 2018 | hs | nn | array | phase |
| solvent | cdcl3 | sspul | y | arraydim | 1024 |
| sample | | PFGflg | y | | |
| ACQUISITION | | hsglvl | 6120 | i | phase |
| sw | 5060.7 | SPECIAL | | 1 | 1 |
| at | 0.150 | temp | not used | 2 | 2 |
| np | 1518 | gain | 44 | | |
| fb | 4000 | spin | 0 | | |
| ss | 32 | GRADIENTS | | | |
| d1 | 1.500 | gzlvl1 | 510 | | |
| nt | 32 | gt1 | 0.001000 | | |
| 2D ACQUISITION | | gzlvl3 | 1530 | | |
| sw1 | 34231.9 | gt3 | 0.001000 | | |
| ni | 512 | gstab | 0.000500 | | |
| phase | arrayed | F2 PROCESSING | | | |
| PRESATURATION | | sb | -0.075 | | |
| satmode | n | sbs | not used | | |
| wet | n | fn | 4096 | | |
| TRANSMITTER | | F1 PROCESSING | | | |
| tn | H1 | gf1 | 0.005 | | |
| sfrq | 599.898 | gfs1 | not used | | |
| tof | -608.8 | proc1 | lp | | |
| tpwr | 58 | fn1 | 2048 | | |
| pw | 9.600 | DISPLAY | | | |
| DECOUPLER | | sp | 533.8 | | |
| dn | C13 | wp | 4334.2 | | |
| dof | 2521.7 | sp1 | 17151.9 | | |
| dm | nnn | wp1 | 8792.0 | | |
| decwave | W40_OnePro-be | rfl | 138.3 | | |
| | | rfp | 0 | | |
| dmf | 35088 | rfl1 | 298.4 | | |
| dpwr | 40 | rfp1 | 0 | | |
| pwxlvl | 59 | PLOT | | | |
| pwx | 9.800 | wc | 250.0 | | |
| HMBC | | sc | 0 | | |
| j1xh | 146.0 | wc2 | 200.0 | | |
| jnxh | 8.0 | sc2 | 0 | | |
| ADIABATCIC | | vs | 10825 | | |
| pwx180ad | OneProbe_-ad300 | th | 2 | | |
| | | ai | av | | |
| pwxlvl180 | 54 | | | | |
| pwx180 | 400.0 | | | | |

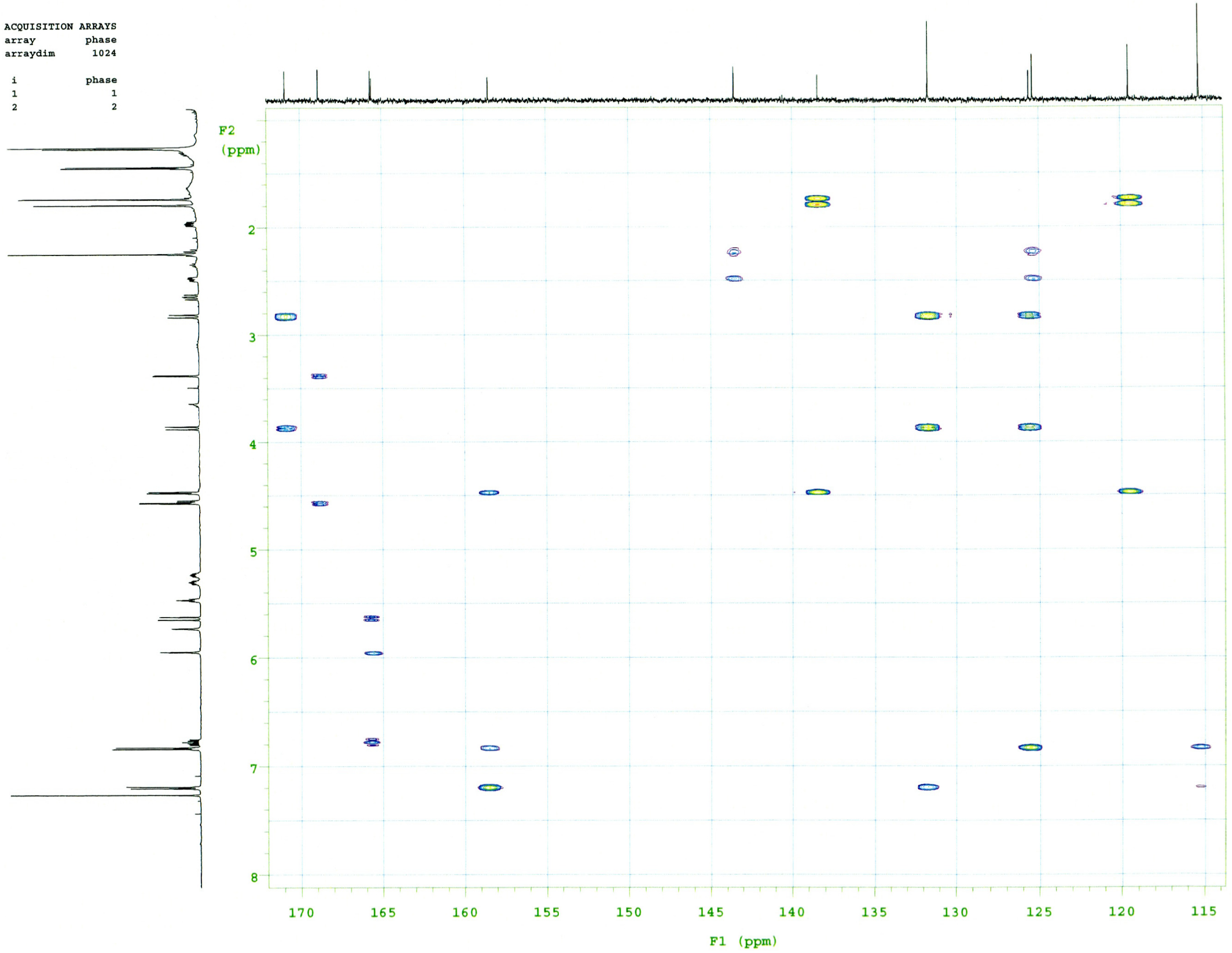

```
135D-4 C26-30

exp56  gHMBCAD

      SAMPLE                FLAGS           ACQUISITION ARRAYS
date   Jan 19 2018  hs              nn     array          phase
solvent       cdcl3  sspul            y     arraydim        1024
sample               PFGflg           y
   ACQUISITION       hsglvl        6120     i              phase
sw           5060.7       SPECIAL           1                  1
at            0.150  temp      not used     2                  2
np             1518  gain            44
fb             4000  spin             0
ss               32      GRADIENTS
d1            1.500  gzlvl1         510
nt               32  gt1       0.001000
  2D ACQUISITION     gzlvl3        1530
sw1         34231.9  gt3       0.001000
ni              512  gstab     0.000500
phase       arrayed     F2 PROCESSING
  PRESATURATION      sb          -0.075
satmode           n  sbs       not used
wet               n  fn            4096
   TRANSMITTER          F1 PROCESSING
tn               H1  gf1          0.005
sfrq        599.898  gfs1      not used
tof          -608.8  proc1           lp
tpwr             58  fn1           2048
pw            9.600       DISPLAY
    DECOUPLER        sp           234.8
dn              C13  wp          4633.2
dof          2521.7  sp1         1707.4
dm              nnn  wp1        10162.6
decwave W40_OnePro-  rf1          138.3
                 be  rfp              0
dmf           35088  rfl1         298.4
dpwr             40  rfp1             0
pwxlvl           59         PLOT
pwx           9.800  wc           250.0
      HMBC           sc               0
j1xh          146.0  wc2          200.0
jnxh            8.0  sc2              0
    ADIABATCIC       vs           10825
pwx180ad OneProbe_~  th               2
              ad300  ai    av
pwxlvl180        54
pwx180        400.0
```

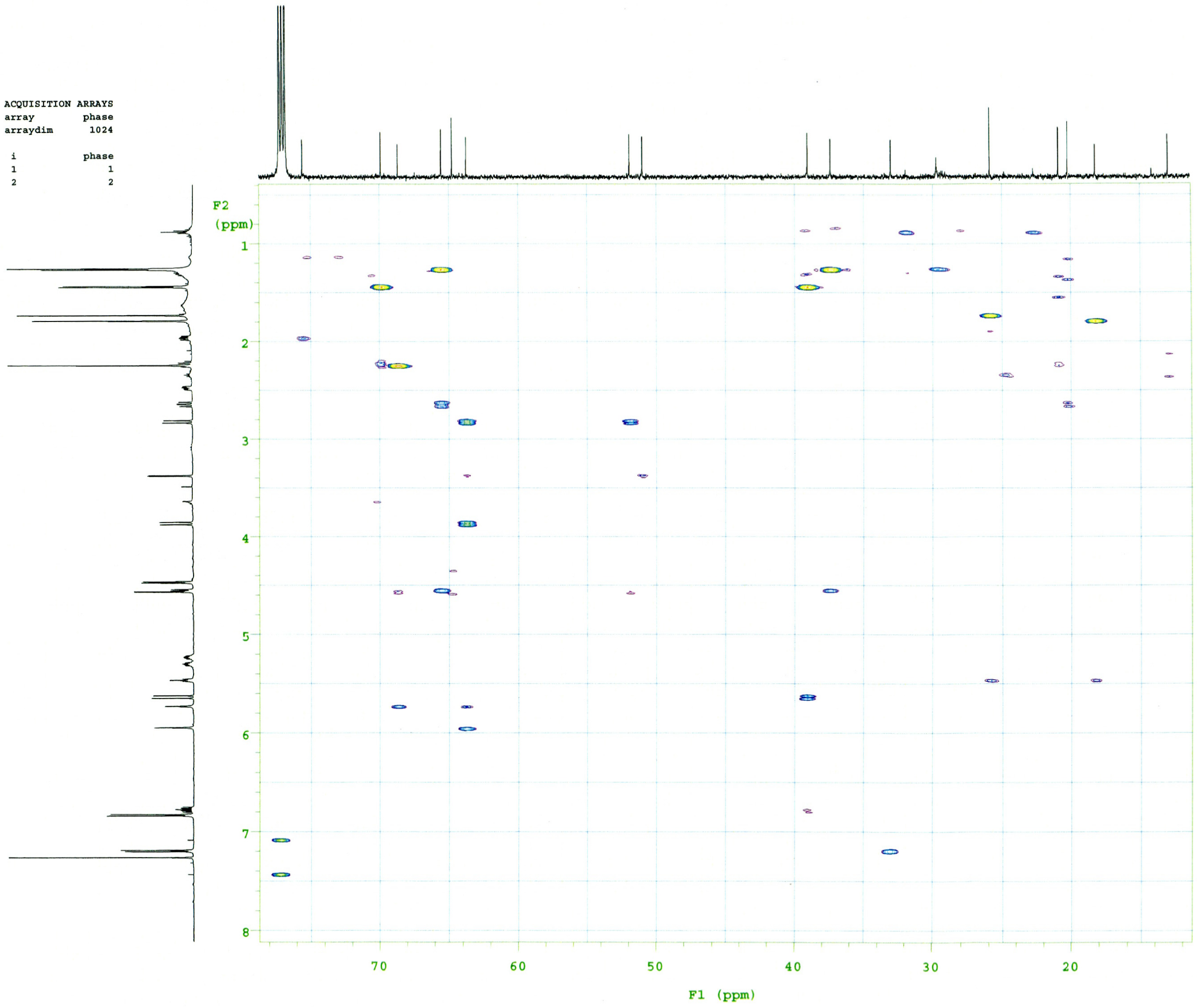

**Figure S8 FABMS of 1**

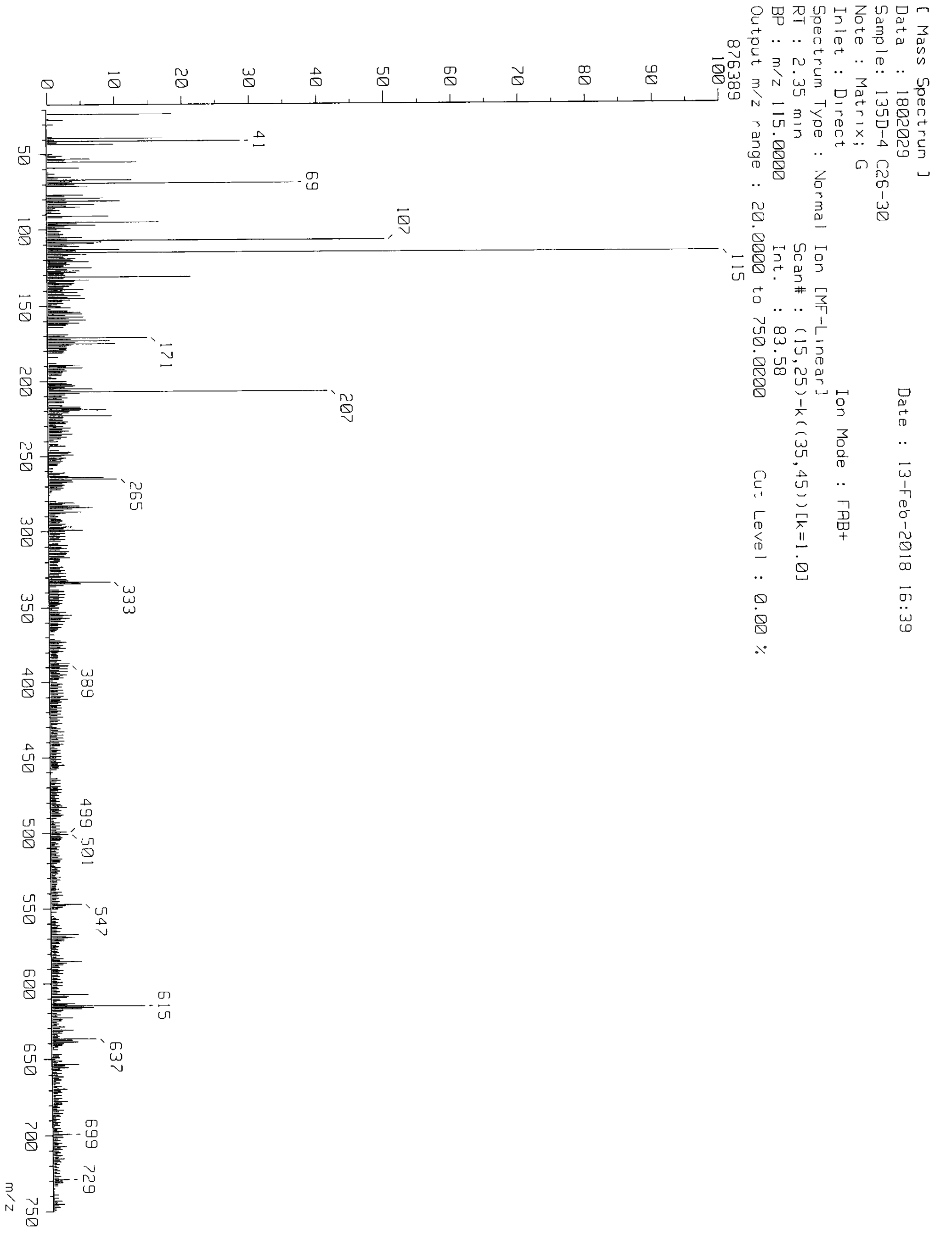

[ Mass Spectrum ] Page: 1
Data : 1802029 Date : 13-Feb-2018 16:39
Sample: 135D-4 C26-30
Note : Matrix; G
Inlet : Direct Ion Mode : FAB+
Spectrum Type : Normal Ion [MF-Linear]
RT : 2.35 min Scan# : (15,25)-k((35,45))[k=1.0]
BP : m/z 115.0000 Int. : 83.58
Output m/z range : 20.0000 to 750.0000 Cut Level : 5.00 %

| m/z | Int. | Norm. |
|---|---|---|
| 23.0000 | 15.50 | 18.55 |
| 39.0000 | 14.38 | 17.20 |
| 41.0000 | 24.00 | 28.71 |
| 43.0000 | 8.28 | 9.91 |
| 53.0000 | 5.29 | 6.33 |
| 55.0000 | 11.09 | 13.26 |
| 67.0000 | 10.49 | 12.56 |
| 69.0000 | 30.62 | 36.64 |
| 71.0000 | 5.01 | 6.00 |
| 77.0000 | 4.49 | 5.37 |
| 79.0000 | 6.95 | 8.32 |
| 81.0000 | 9.01 | 10.78 |
| 83.0000 | 6.02 | 7.20 |
| 91.0000 | 7.60 | 9.09 |
| 95.0000 | 13.92 | 16.65 |
| 97.0000 | 5.98 | 7.15 |
| 105.0000 | 4.30 | 5.14 |
| 107.0000 | 41.92 | 50.16 |
| 108.0000 | 6.82 | 8.16 |
| 109.0000 | 5.78 | 6.92 |
| 113.0000 | 8.97 | 10.73 |
| 115.0000 | 83.58 | 100.00 |
| 116.0000 | 4.82 | 5.77 |
| 121.0000 | 5.12 | 6.12 |
| 125.0000 | 5.50 | 6.59 |
| 131.0000 | 17.81 | 21.31 |
| 133.0000 | 5.10 | 6.11 |
| 139.0000 | 4.43 | 5.30 |
| 145.0000 | 4.61 | 5.52 |
| 155.0000 | 4.26 | 5.10 |
| 157.0000 | 4.42 | 5.28 |
| 159.0000 | 4.71 | 5.64 |
| 171.0000 | 12.34 | 14.77 |
| 173.0000 | 7.68 | 9.19 |
| 175.0000 | 8.42 | 10.07 |
| 191.0000 | 4.30 | 5.14 |
| 205.0000 | 5.51 | 6.59 |
| 207.0000 | 34.68 | 41.49 |
| 219.0000 | 7.16 | 8.57 |
| 223.0000 | 7.84 | 9.38 |
| 264.0000 | 6.79 | 8.12 |
| 265.0000 | 8.44 | 10.10 |
| 284.0000 | 5.38 | 6.44 |
| 333.0000 | 7.52 | 9.00 |
| 607.0000 | 4.53 | 5.42 |
| 615.0000 | 11.56 | 13.83 |
| 616.0000 | 5.19 | 6.21 |
| 637.0000 | 5.49 | 6.57 |

[ Elemental Composition ]                                                    Page: 1
Data : 1802049                    Date : 14-Feb-2018 18:38
Sample: 135D-4 C26-30
Note : Matrix; G
Inlet : Direct                    Ion Mode : FAB+
RT : 8.25 min                     Scan#: (57,62)
Elements : C 34/28, H 45/35, N 4/0, O 11/0, S 1/0
Mass Tolerance : 20ppm, 1mmu if m/z > 50
Unsaturation (U.S.) : -1.0 - 30.0

| Observed m/z | Int% | Err[ppm / mmu] | U.S. | Composition |
|---|---|---|---|---|
| 615.2380 | 100.0 | +0.7 / +0.4 | 14.5 | C 31 H 39 N 2 O 9 S |

**Figure S9 IR spectrum of 1**

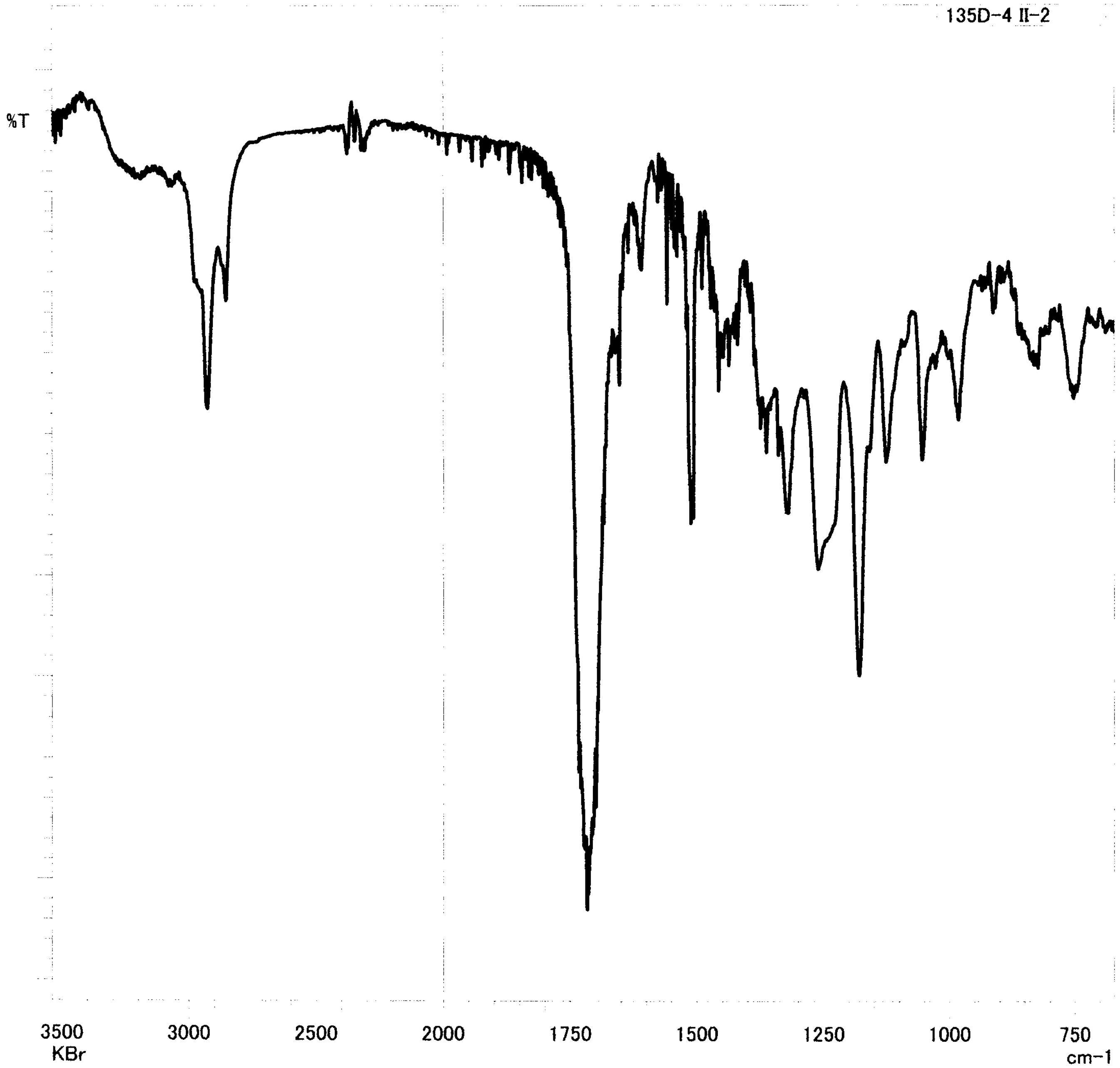

Supplement: Supplementary file 1 [file marinedrugs-18-00320-s001.pdf]
